# Molecular basis for acyl carrier protein–ketoreductase interaction in *trans*-acyltransferase polyketide synthases†

**DOI:** 10.1039/d1sc03478b

**Published:** 2021-09-30

**Authors:** Munro Passmore, Angelo Gallo, Józef R. Lewandowski, Matthew Jenner

**Affiliations:** Department of Chemistry, University of Warwick Coventry CV4 7AL UK m.jenner@warwick.ac.uk; Warwick Integrative Synthetic Biology Centre (WISB), University of Warwick Coventry CV4 7AL UK

## Abstract

The biosynthesis of polyketides by type I modular polyketide synthases (PKS) relies on co-ordinated interactions between acyl carrier protein (ACP) domains and catalytic domains within the megasynthase. Despite the importance of these interactions, and their implications for biosynthetic engineering efforts, they remain poorly understood. Here, we report the molecular details of the interaction interface between an ACP domain and a ketoreductase (KR) domain from a *trans*-acyltransferase (*trans*-AT) PKS. Using a high-throughput mass spectrometry (MS)-based assay in combination with scanning alanine mutagenesis, residues contributing to the KR-binding epitope of the ACP domain were identified. Application of carbene footprinting revealed the ACP-binding site on the KR domain surface, and molecular docking simulations driven by experimental data allowed production of an accurate model of the complex. Interactions between ACP and KR domains from *trans*-AT PKSs were found to be specific for their cognate partner, indicating highly optimised interaction interfaces driven by evolutionary processes. Using detailed knowledge of the ACP:KR interaction epitope, an ACP domain was engineered to interact with a non-cognate KR domain partner. The results provide novel, high resolution insights into the ACP:KR interface and offer valuable rules for future engineering efforts of biosynthetic assembly lines.

## Abstract 2

The interaction epitope between a cognate KR–ACP domain pairing from a *trans*-AT polyketide synthase is elucidated in molecular detail, providing unique insights into recognition and specificity of the interface.

## Graphical abstract

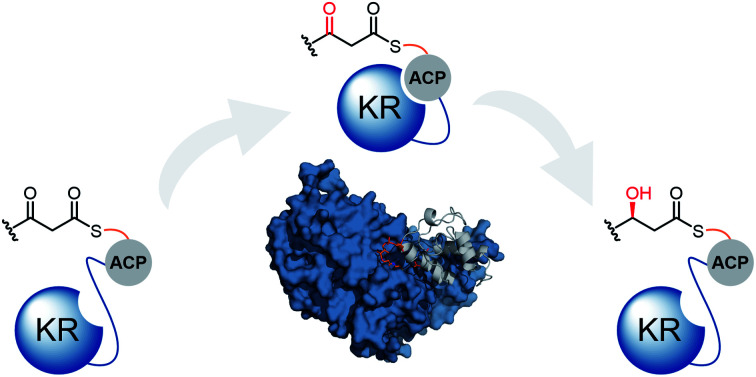

## Introduction

Polyketides constitute a valuable family of natural products, many of which find application in both medicine and agriculture.^1,2^ Despite extraordinary structural complexity and diversity, the chemical logic underpinning their biosynthesis is elegantly simple; the head-to-tail decarboxylative condensation of malonyl and acyl units to generate linear carbon frameworks, upon which additional structural diversification can be applied.^3^ In bacteria, polyketides are typically biosynthesised by type I modular polyketide synthases (PKSs). These multi-domain enzymes conform to a paradigm of covalent substrate attachment for exceptional processivity, and as a result, are often likened to molecular assembly lines. A co-enzyme A (CoA)-derived phosphopantetheine (Ppant) moiety, post-translationally attached to the acyl carrier protein (ACP) domains by a 4′-phosphopantetheinyl transferase (PPTase), serves to tether biosynthetic intermediates to the ACP domain *via* a thioester linkage.^4^

**Fig. 1:**
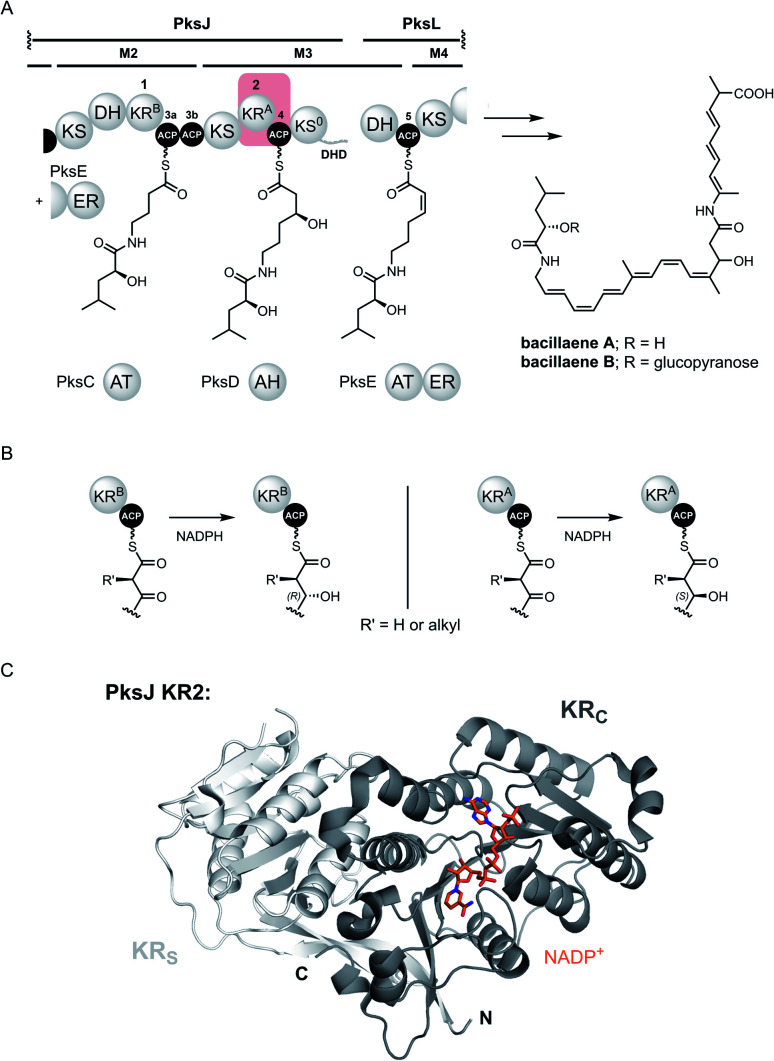
(A) Partial domain and module organisation of the *trans*-AT PKS responsible for production of bacillaenes, showing the proposed structure of each ACP-bound thioester intermediate. KR and ACP domains have been numbered in a sequential manner, and tandem ACPs are given lower case alphabetical subscripts. The KR2 and ACP4 domains that form the basis for this study are highlighted with a red box. Domain abbreviations are as follows: KS, ketosynthase; KS^0^, non-elongating ketosynthase; KR, ketoreductase; DH, dehydratase; ER, enoylreductase (*trans*-acting); DHD, dehydratase-docking domain; ACP, acyl carrier protein. (B) Formation of ACP-bound (3*R*)-3-hydroxy (left) and (3*S*)-3-hydroxy (right) thioester intermediates by type B and type A KR domains, respectively. (C) Crystal structure of PksJ KR2 domain with NADP^+^ bound (PDB: 5KTK). The structural domain (KR_S_) is coloured in light grey and the catalytic domain (KR_C_) is coloured in dark grey.

The modular PKS architecture is defined by repeating units of catalytic domains, akin to that of fatty acid biosynthesis, which catalyse a single cycle of chain extension and subsequent α/β-carbon modification. Within a module, an acyl transferase (AT) domain loads an (alkyl)malonyl-derived extender unit onto the ACP domain, allowing the ketosynthase (KS) domain to catalyse a Claisen-like condensation with the upstream polyketide chain yielding a β-keto thioester intermediate. Optional processing domains in the module affords additional diversity at the α- and β-carbon positions of the resulting β-keto thioester. These include C-methyltransferase (MT) domains, which methylate the α-carbon, in addition to ketoreductase (KR), dehydratase (DH), and enoylreductase (ER) domains that generate hydroxyl, olefinic and fully saturated intermediates, respectively.^5–7^ Evolution has given rise to two discrete classes of modular PKS: *cis*-AT and *trans*-AT. Whilst the *cis*-AT PKSs have AT domains integrated into each module; the *trans*-AT (or AT-less) PKSs employ a standalone AT domain to supply malonyl extender units to all ACP domains in the assembly line.^8–10^ In addition to lacking an integrated AT domain, the *trans*-AT PKSs have other characteristic features, these include modules that are split across subunits, non-elongating KS domains and an assortment of other *trans*-acting catalytic domains.^11^ The archetypal *trans*-AT PKS responsible for the biosynthesis of bacillaene highlights some of these aberrant features (Fig. 1A).

Intra-molecular interactions between the ACP domain and catalytic domains play a pivotal role in ensuring biosynthetic fidelity in both *cis*- and *trans*-AT PKSs.^12,13^ Within a module, the ACP domain must shuttle biosynthetic intermediates between catalytic domains in an ordered manner, engaging in specific protein–protein interactions with each domain.^14^ Although the natural tethering of PKS domains means that the effective local concentration of the ACP domain is high,^15^ as a consequence, the interactions with catalytic domains are often low affinity, making them difficult to characterise experimentally. This has resulted in the intra-molecular interaction network between ACP domains and catalytic domains remaining largely unexplored, with the majority of our current knowledge limited to *cis*-AT PKS systems. Of particular note are a set of cryo-EM structures from an entire *cis*-AT module, which capture the various substrate-bound forms of the ACP domain interfacing with the KS, AT and KR domains.^16,17^ Although this provided remarkable insights into the positioning of the ACP domain during the catalytic cycle, precise residue-level information regarding each interface is obscured by the resolution of the technique at the time. The development of mechanism-based crosslinking has allowed certain ACP domain-mediated interactions to be covalently ‘trapped’, permitting crystallisation of these complexes.^18^ To date, this approach has yielded high-resolution structures for various FAS and PKS systems,^19–22^ however, structures of crosslinked ACP-catalytic domain complexes from modular PKSs have proved more challenging. It should be noted, that although crosslinkers have been developed to target most catalytic domains, probes for trapping complexes with the KR and ER domain are yet to be established.

Whilst all ACP domain-mediated interactions drive the biosynthetic process, the interface between the ACP domain and the KR domain is of particular importance from a stereochemical perspective. Here, the ACP domain delivers a β-keto thioester intermediate to the KR domain, which catalyses ketoreduction using the 4-*pro-S* hydride of NADPH to yield a β-hydroxy thioester product.^23^ The stereochemical outcome of this reaction is controlled by the nature of the KR domain: ‘type A’ (KR^A^) and ‘type B’ (KR^B^) domains generate (3*S*) and (3*R*)-configured 3-hydroxy thioester products, respectively, information that can be obtained by inspection of the primary sequence (Fig. 1B).^24–26^ Structurally, KR domains are monomeric and consist of an N-terminal structural subdomain (KR_S_), and a C-terminal catalytic subdomain (KR_C_) which binds NADPH; both of which exhibit a Rossmann-like fold (Fig. 1C).^27^ Numerous structures of excised KR domains from modular PKS systems have been reported in recent years, primarily to understand the factors involved in stereocontrol.^28^ However, the molecular basis for their interaction with the ACP domain remains poorly understood, particularly for *trans*-AT PKS systems.

Herein, we apply a highly complementary set of techniques to elucidate residue-level details underpinning the interaction interface between a cognate pair of ACP (PksJ ACP4) and KR (PksJ KR2) domains from module 3 of the bacillaene *trans*-AT PKS (Fig. 1A). Our data establish the location and specific binding epitopes for the interface, allowing a data-driven model of the ACP:KR complex to be produced. Unlike *cis*-AT PKSs,^29,30^ interactions between cognate pairs of ACP and KR domains from *trans*-AT PKSs were found to be highly specific, and the molecular basis for this specificity is rationalised though a detailed understanding of the interaction epitope. This knowledge is then applied to engineer an ACP domain to interact with a non-cognate KR domain, highlighting the bioengineering potential of the *trans*-AT PKS systems.

## Results and discussion

### Ketoreduction assay to examine PksJ ACP4:KR2 interactions

**Fig. 2:**
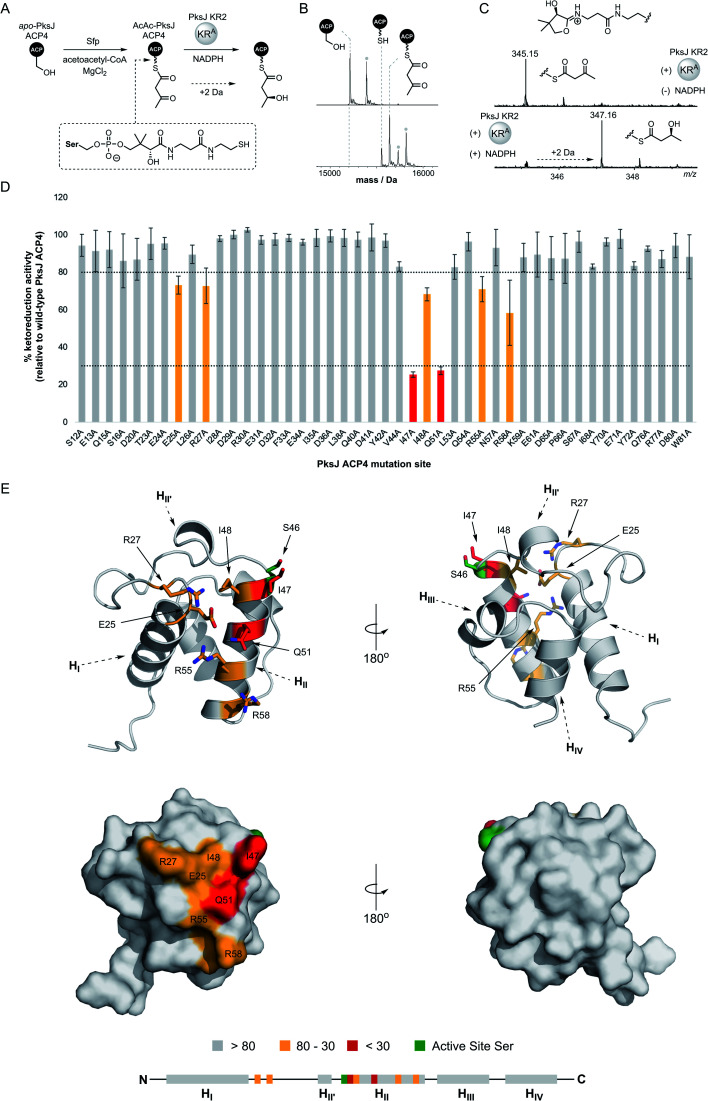
(A) Schematic overview of ketoreduction assay used to probe ACP:KR interactions. (B) Deconvoluted mass spectra of *apo*-PksJ ACP4 domain (top) and its conversion to the 3-ketobutyryl-ACP4 species (bottom). The identity of each species is highlighted by dotted lines. The grey dot indicates the typical post-translational gluconylation of the His–Tag from pET28a(+) recombinant proteins. (C) Mass spectra of ketoreduction assays monitored by 4′-phosphopantetheine ejection. In the absence of the NADPH co-factor (top), only the 3-ketobutyryl-ACP4 species is observed (Ppant ion *m*/*z* 345.15). Addition of the NADPH co-factor results in complete conversion to the (3*S*)-hydroxybutyryl-ACP species (Ppant ion *m*/*z* 347.16). (D) Bar chart showing PksJ KR2-catalysed ketoreduction activity for all alanine-mutants of PksJ ACP4. The values are expressed as a percentage of WT PksJ ACP4 activity after 5 min incubation. Arbitrary cut-offs bounds of 80% and 30% ketoreduction activity are depicted as dashed lines and highlighted in grey (>80%), orange (80–30%) and red (<30%). Error bars represent ±standard deviation from the mean, where *n* = 3. (E) Relative ketoreduction activities of alanine-mutants mapped onto a homology model of PksJ ACP4 domain and coloured according to the accompanying bar chart. Cartoon (top) and surface (bottom) representations are shown. Residues in the 80–30% and <30% bounds are highlighted in stick representation. A linear schematic of PksJ ACP4 secondary structure elements is displayed, highlighting positions of residues critical to the PksJ ACP4:KR2 interaction.

In the first instance, excised PksJ ACP4 and PksJ KR2 domains were overproduced in *Escherichia coli* as N-terminal pHis_6_ fusion proteins, purified to near-homogeneity using immobilized metal-ion affinity chromatography (IMAC), and analysed by intact protein mass spectrometry (MS) to confirm their identity (ESI Fig. S1†). To generate a substrate mimic for the PksJ KR2 domain, the *apo*-PksJ ACP4 domain was converted to a 3-keto-butyryl-PksJ ACP4 species using the PPTase, Sfp, and acetoacetyl-coenzyme A (Fig. 2A). Conversion to the 3-keto-butyryl-PksJ ACP4 form was verified by intact protein MS analysis (Fig. 2B). Previous work has shown that the PksJ KR2 domain converts a 3-keto-pentanoyl-*N*-acetyl cysteamine thioester substrate to a (3*S*)-3-hydroxy-pentanoyl thioester product, yielding the expected stereochemistry for the A-type family of KR domains.^31^ Building on this, an MS-based assay was devised to monitor conversion of 3-keto-butyryl-PksJ ACP4 to (3*S*)-3-hydroxy-butyryl-PksJ ACP4 catalysed by the PksJ KR2 domain, thereby forming an experimental platform to probe interactions between the domains.

Due to the small increase in mass (+2 Da) associated with PksJ KR2-catalysed conversion of 3-keto-butyryl-PksJ ACP4 to (3*S*)-3-hydroxy-butyryl-PksJ ACP4, we elected to monitor ejected phosphopantetheinyl ions from the intact PksJ ACP4 protein.^32^ This allowed an unambiguous mass shift associated with ketoreduction to be identified, yielding ions at *m*/*z* = 345.15 and 347.16 corresponding to the 3-keto-butyryl and (3*S*)-3-hydroxy-butyryl species, respectively (Fig. 2C). Initial experiments revealed that PksJ KR2 was able to catalyse complete conversion of 3-keto-butyryl-PksJ ACP4 to (3*S*)-3-hydroxy-butyryl-PksJ ACP4 in the presence of NADPH after a 10 min incubation period. Time-resolved analysis of the reaction showed near-complete conversion of 3-keto-butyryl-PksJ ACP4 to (3*S*)-3-hydroxy-butyryl-PksJ ACP4 after 5 min and was used as the time-point at which to quench the reaction, in order to observe changes in reaction efficiency as a result of mutations (ESI Fig. S2†).

### Mapping the KR-binding epitope of PksJ ACP4 using alanine scanning mutagenesis

Having established a functional assay, we elected to map the KR-binding epitope of PksJ ACP4 using alanine scanning mutagenesis, a technique that has been shown to be an effective for the identification of interaction sites on the surface of carrier proteins.^33,34^ In order to predict surface exposed residues of PksJ ACP4, a homology model was constructed using the iTASSER server^35^ and the *apo*-MmpA ACP–ACP di-domain as a template^36^ (PDB: 2L22). Following analysis of the model, a total of 50 surface-exposed residues were then individually mutated to Ala, overproduced, and purified to homogeneity in their *apo*-form. It is worth noting that five of these mutants (W17A, F39A, D45, L64A, T74A) could not be expressed in a soluble form and were therefore excluded from the study. The remaining 45 X → Ala mutants resulted in ∼95% surface coverage of the solvent exposed non-Gly/Ala residues from the core 4α-helix bundle of the PksJ ACP4 domain (ESI Fig. S3†). These mutants were enzymatically converted to their 3-keto-butyryl form and analysed by intact protein MS to confirm the modification. All of the Ala-mutants achieved levels of modification comparable to that of wild-type (WT) PksJ ACP4 domain (Fig. 2B), indicating that none of the X → Ala mutations significantly perturbed Sfp-mediated phosphopantetheinylation.

The suite of 3-keto-butyryl-loaded PksJ ACP4 Ala-mutants were then subjected to the MS-based ketoreduction assay to assess the impact of each residue on the PksJ ACP4:KR2 interaction interface. The extent of ketoreduction was measured as a ratio of the Ppant ejection ions corresponding to the 3-keto-butyryl and (3*S*)-3-hydroxy-butyryl species, and then expressed as a percentage of the WT PksJ ACP4:KR2 reaction (Fig. 2D). The majority of Ala-mutants displayed an activity profile similar to that of WT PksJ ACP4, with relative ketoreduction activity >80%, indicating that mutation of these residues to Ala does not perturb interaction with PksJ KR2 domain. However, seven Ala-mutants (E25A, R27A, I47A, I48A, Q51A, R55A, R58A) exhibited a significant reduction in ketoreduction activity compared to the WT (<80% activity). Of these mutants, I47A and Q51A resulted in <30% activity (Fig. 2D and ESI Fig. S4†). Circular dichroism (CD) spectra of these seven Ala-mutants were near-identical to that of WT PksJ ACP4 domain, suggesting no noticeable alterations to secondary structure elements or unfolding upon mutation, meaning that diminished activity must be due to removal of important side chain functionality at the ACP:KR interface (ESI Fig. S5†). This provided clear evidence that the seven residues identified contribute to the PksJ ACP4:KR2 interface, although each to differing extents.

Positions of KR-interacting residues identified by alanine scanning mutagenesis were then mapped onto the PksJ ACP4 domain homology model to visualise the binding epitope (Fig. 2E). The resulting heat map revealed a clustering of critical residues in close proximity to the active site serine (S46), to which the Ppant arm is attached. Residues I47, I48, Q51, R55 and R58 are all situated on helix II (H_II_), with I47 and I48 directly adjacent to S46. The Q51, R55 and R58 residues are located at sequential turns running down H_II_, indicating the importance of the residues displayed on the surface of this helix. Interestingly, residues E25 and R27 are located on the loop region connecting H_I_ and H_II_, and therefore distant at the sequence level, but are close in three-dimensional space to the critical residues on H_II_, contributing to the well-defined binding epitope (Fig. 2E).

### Mapping the ACP-binding interface of PksJ KR2 using carbene footprinting

We next sought to establish residues on the PksJ KR2 domain which contribute to complex formation with the PksJ ACP4 domain. To achieve this, we employed the recently developed carbene footprinting methodology; a structural mass spectrometry technique which exploits covalent labelling of solvent-exposed residues on a protein surface using a reactive carbene species, formed by *in situ* photolysis of the corresponding diazirine.^37,38^ Proteolytic digest and subsequent LC-MS analysis provides peptide-level information of the extent of covalent labelling. Conducted in the presence and absence of a binding partner, differential labelling of peptides can be observed allowing binding sites (solvent excluded, masked) and conformational changes (solvent accessible, unmasked) to be identified.

Firstly, a PksJ KR2-ACP4 di-domain construct was prepared, overproduced in *E. coli* as an N-terminal pHis_6_ fusion protein and purified to near-homogeneity (ESI Fig. S1†). Covalent tethering of the KR and ACP domains increases the localised concentration of each domain, thus promoting complex formation and facilitating binding site identification. The di-domain construct encompasses the full sequence of the PksJ ACP4 domain used in the biochemical assays, and also incorporates a 24-residue linker region between the two domains, which enhances activity as a result of increased effective local concentration. A time-course ketoreduction assay of PksJ KR2–ACP4 conducted under the same conditions as the isolated PksJ KR2 + PksJ ACP4 domain system showed the increased efficiency of the reaction in the covalently tethered construct (ESI Fig. S6†), in agreement with previous observations.^39^

**Fig. 3:**
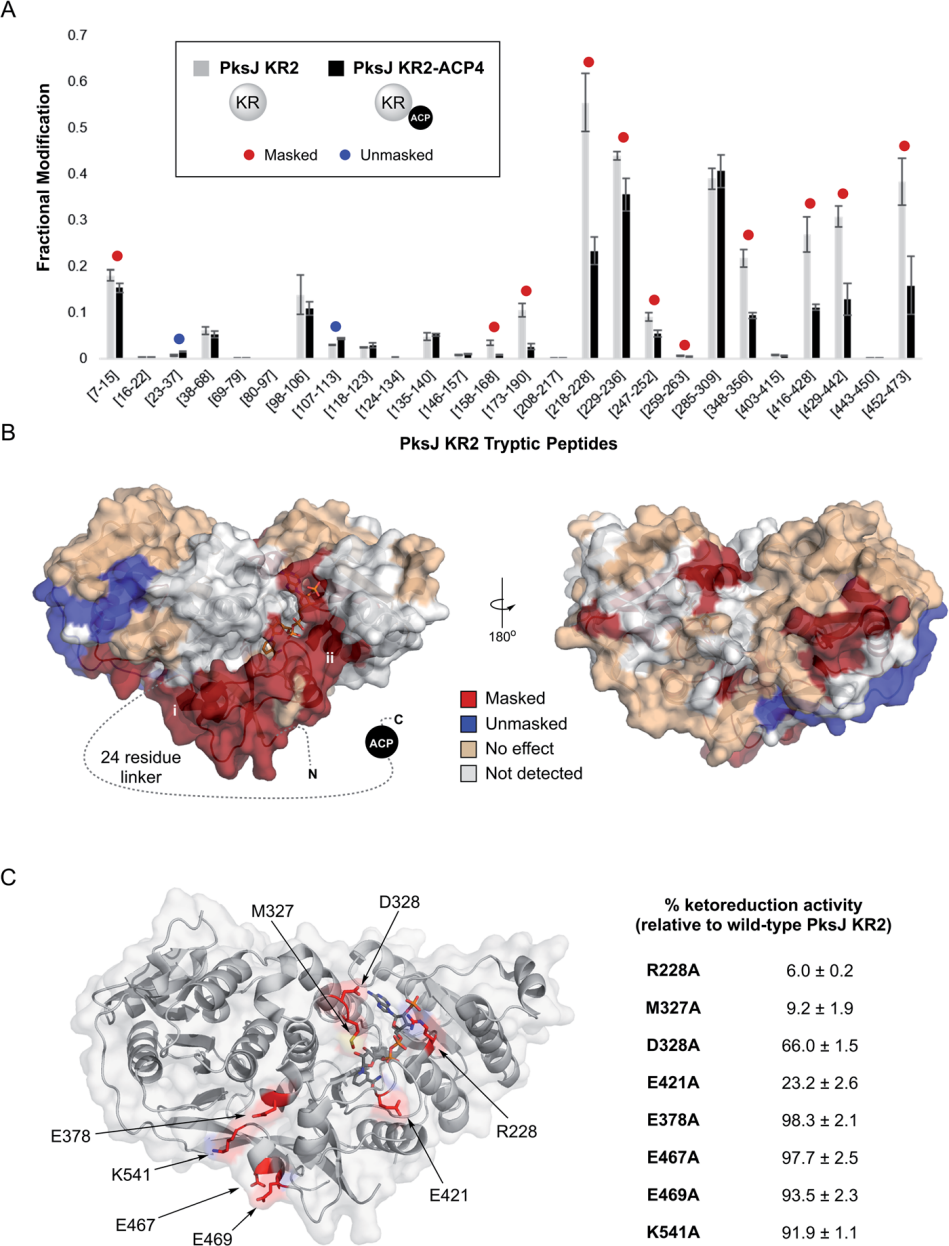
(A) Fractional modification of PksJ KR2 tryptic peptides arising from photochemical labelling in the absence (grey bars) and presence (black bars) of PksJ ACP4 domain (covalently tethered *via* PksJ KR2-ACP4 di-domain construct). Error bars represent ±standard deviation from the mean, where *n* = 3. Significant differences (Student's *t*-test, *P* < 0.05) are highlighted with a red (masked) or blue (unmasked) dot. (B) Structure of PksJ KR2 (from PDB: 5KTK) showing the locations of masked (red), unmasked (blue) and unaffected (wheat) peptide regions in the presence of PksJ ACP4 domain. A dotted line represents the 24-residue linker region that connects the KR domain to the ACP domain in the N- to C-terminal direction. The two plausible binding sites for the ACP domain based on masking are highlighted with roman numerals. (C) Structure of PksJ KR2 with residues on the surface hypothesised to be involved in binding PksJ ACP4 highlighted in red. Residues were hypothesised to be important based on carbene footprinting data (shown above), and preliminary docking studies. Relative ketoreduction activities of X → Ala mutations of identified residues are shown, and the values are expressed as a percentage of WT PksJ KR2 activity after 5 min incubation. Errors are ±standard deviation from the mean, where *n* = 3.

Carbene footprinting experiments were carried out on solutions containing PksJ KR2 domain and *apo*-PksJ KR–ACP di-domain followed by LC-MS analysis of the tryptic digests, which yielded 67.4% sequence coverage of the PksJ KR2 domain (ESI Fig. S7†). Differential labelling was observed for 13 tryptic peptides; 11 of which exhibited reduced labelling (masking), and 2 increased labelling (unmasking) in the presence of the ACP domain and associated linker region (Fig. 3A). The results of these experiments were then visualised on the crystal structure of the PksJ KR2 domain (PDB: 5KTK) (Fig. 3B).^31^ This revealed an extended region of masking on the PksJ KR2 domain surface, running from the KR_S_ subdomain where the 24-residue linker emanates, across to the KR_C_ subdomain where the NADPH co-factor is bound. Interestingly, the presence of the PksJ ACP4 domain and associated linker region also resulted in unmasking within the KR_S_ subdomain (peptides: Q23–R37 and L107–K113), suggesting a conformational change that increases solvent exposure in this region, possibly to accommodate part of the 24-residue linker (Fig. 3B). Given the requirement of NADPH for catalysis, we also conducted the footprinting experiment with NADPH bound to the KR domain to examine whether this changed the differential labelling profile upon binding of the ACP domain. Here, the Q23–R37 peptide, which became unmasked upon ACP binding in the absence of NADPH (Fig. 3A), appears to experience no effect upon ACP binding when NADPH is bound (ESI Fig. S8†). This suggests that NADPH may provide some additional overall stability to the KR domain when bound, preventing movement in this region. All other peptides maintained the same differential labelling profile, indicating that the interface is largely similar in the NADPH-bound and unbound states.

The carbene footprinting data indicated two plausible sites of interaction for the PksJ ACP4 domain on the PksJ KR2 domain surface: (i) next to the KR_S_ subdomain situated at the open side of the substrate binding channel or (ii) at the KR_C_ subdomain close to the NADPH co-factor binding site. Furthermore, guided by our experimental data, analysis in ChimeraX virtual reality^40^ and preliminary docking using high ambiguity driven biomolecular docking (HADDOCK)^41^ suggested that both sites had the potential to be viable solutions for the PksJ KR2:ACP4 complex. We therefore opted to mutate surface exposed residues at each site, and monitor the effect using our MS-based ketoreduction assay to identify the site of PksJ ACP4 complex formation. Using information from carbene footprinting and preliminary docking simulations, four residues at each site were individually mutated to alanine: E378, E467, E469 and K541 from the site adjacent to the KR_S_ subdomain; and R228, M327, D328 and E421 from the site located near the NADPH binding site on the KR_C_ subdomain. It should be noted that although residues M327, D328 and E378 are located in peptides not detected in carbene footprinting experiments (ESI Fig. S7†), the preliminary docking solutions suggested that they play an important role at the interface. While mutations to residues near the KR_S_ subdomain had a negligible effect on ketoreduction activity (92–98% of WT PksJ KR2), a significant decrease in activity was observed for all mutants at the KR_C_ subdomain site (6–66% of WT PksJ KR2) (Fig. 3C). Based on the structure of PksJ KR2, the side chain of R228 is situated in a loop region involved in co-factor binding, and forms salt bridge interactions with the phosphate groups of NADPH.^31^ It is therefore conceivable that some of the reduction in catalytic activity in the R228A mutant may be due to a reduction in co-factor affinity. However, the side chains of M327, D328 and E421 are not involved in co-factor binding and likely contribute directly to the interface with the PksJ ACP4 domain. These mutagenesis experiments suggested that the masking pattern observed in the carbene footprinting experiments is consistent with docking of the PksJ ACP4 domain at the KR_C_ subdomain site, and the additional masking occurred as a result of the 24-residue linker region extending across the KR domain surface,^42^ as depicted in Fig. 3B. Interestingly, the ACP-docking site identified on the PksJ KR2 domain is consistent with data obtained for KR:ACP interface in the pikromycin *cis*-AT PKS using single particle cryo-EM^17^ (ESI Fig. S9†), suggesting that a similar ACP-docking site is employed by KR domains across *cis*- and *trans*-AT PKS systems.

### Computational docking and validation of the PksJ ACP4:KR2 complex

**Fig. 4:**
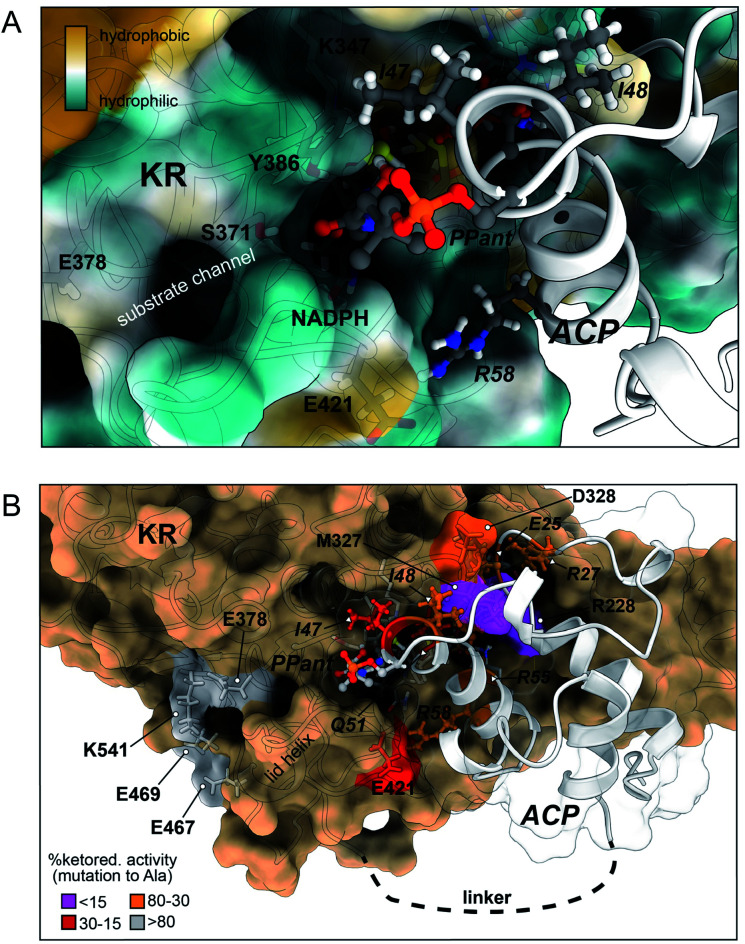
Docked model of the *holo*-PksJ ACP4 domain–PksJ KR2 domain complex. The crystal structure of PksJ KR2 domain (PDB: 5KTK) and a homology model of PksJ ACP4 were used, with residues identified from alanine-scanning mutagenesis and carbene footprinting experiments employed as constraints for the docking simulations. (A) Close-up view of the Ppant arm entering the substrate channel of PksJ KR2. A distance of ∼8 Å from the Ppant thiol to the NADPH 4-*pro-S* hydride is achieved, allowing space for a 3-ketoacyl thioester intermediate to adopt a catalytically relevant conformation. The surface of the PksJ KR2 domain is coloured according to hydrophilic/hydrophilic properties, and key residues involved in the interaction and catalysis are labelled. (B) Full perspective of the docked complex. Relative ketoreduction activities of alanine-mutants are mapped onto the respective structures and coloured according to the effect each X → Ala mutation: <15% (magenta), 15–30% (red), 30–80% (orange) and >80% (grey). The 200 ns MD trajectory stripped of water and ions with frames every 1 ns is available for download from Mendeley Data DOI: 10.17632/8shpf4mrs6.1.

Taken together, the experimental data from alanine scanning mutagenesis and carbene footprinting provided near-residue level resolution of the PksJ ACP4:KR2 interaction interface. As a result, we elected to utilise this information as restraints for docking simulations using HADDOCK to produce a model based on our experimental observations. Applying this approach, a series of models of the PksJ KR2:ACP4 complex were generated using the crystal structure of PksJ KR2 (PDB: 5KTK) and the homology model of *apo*-PksJ ACP4 as input structures. The cluster of solutions with the highest score showed excellent agreement with the experimental data, which positioned the PksJ ACP4 Ppant attachment site (S46) at the opening of the NADPH-binding channel, ∼22 Å from the 4-*pro-S* hydride of NADPH. A Ppant arm was then manually modelled into the structure using Chimera X virtual reality and the complex was energy minimised, followed by a 200 ns classical molecular dynamics simulation in AMBER. The model shown in Fig. 4 is based on a frame extracted at 600 ps of MD simulation places the Ppant thiol ∼8 Å from the 4-*pro-S* hydride, allowing sufficient space for the 3-ketoacyl biosynthetic intermediate to enter the active site and position the C3 keto group proximal to the 4-*pro-S* hydride of NADPH and the catalytic tyrosine (Y386) residue. The rest of the polyketide intermediate likely sits in the largely hydrophobic substrate binding channel, running away from the NADPH co-factor (Fig. 4A), though further opening of the channel (observed during the MD) might be required to fully accommodate the native substrate.

The docked model places many of the residues identified by alanine scanning mutagenesis in critical positions at the interaction interface and begins to explain our experimental observations. Examination of the docked complex finds M327 on the PksJ KR2 domain accommodated into a hydrophobic groove on the PksJ ACP domain (Fig. 4B and ESI Fig. S10A†). Mutation of M327 dramatically reduced ketoreduction activity (Fig. 3C), probably as a result of ordered water molecules able to occupy the interface in the absence of an aliphatic side chain. The I48 residue on the PksJ ACP4 domain contributes to the hydrophobic interface with M327, and together with I47 also serves to plug a hydrophobic hole on the PksJ KR2 domain surface (Fig. 4B and ESI Fig. S10B†). Positioned by I48, the I47 residue appears to play an important role in guiding the Ppant arm into the substrate binding channel, and mutation of both these residues severely diminished ketoreduction activity (Fig. 2D and E). The model also highlights that D328 on the PksJ KR2 domain is pulled away from the hydrophobic interface by an intra-domain interaction with R342, which explains the relatively modest reduction in activity upon mutation to Ala (Fig. 3C and ESI Fig. S10A†). Mutation of R228 on the PksJ KR2 domain reduced ketoreduction activity considerably (Fig. 3C), and the docked complex indicates that it forms critical charged contacts with E25 on the PksJ ACP4 domain (Fig. 4B and ESI Fig. S10C†). The E25 residue is spatially positioned through an intra-domain contact with R27 (ESI Fig. S10C†), and mutation of either of these residues diminished ketoreduction activity (Fig. 2D and E).

Interestingly, the docked complex suggests that the positively charged R55 interacts directly with one of the phosphate groups forming part of the phosphodiester linkage of NADPH, with the orientation of R55 dictated *via* an intra-domain interaction with Q51 (ESI Fig. S10D†). The mutation of both these residues significantly perturbed ketoreduction activity (Fig. 2D and E). This observation implies that some interactions between the ACP and KR domains may only be possible when NADPH is bound and could be the basis for controlling complexation between the two domains, and by extension catalytic activity. During the time-course of the MD simulations, E421 was observed to interact with R58 on the PksJ ACP4 domain in addition to an intra-domain interaction with K415. Interaction between E421 and K415 appears to stabilise a connecting loop region, which provides a platform for the PksJ ACP4 domain to dock correctly (ESI Fig. S10E†). Taken together, these observations likely explain the reduction in activity observed upon mutation of E421 on the PksJ KR2 domain and R58 on the PksJ ACP4 domain (Fig. 2D and 3C).

### Probing the specificity of ACP:KR interactions in *trans*-AT PKSs

**Fig. 5:**
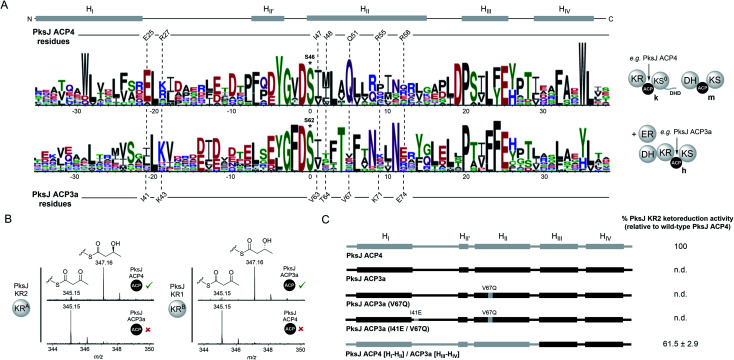
(A) Sequence logos for *trans*-AT PKS ACP domains from different phylogenetic clades: ‘clade k’ (top, *e.g.* PksJ ACP4), and ‘clade h’ (bottom, *e.g.* PksJ ACP3a). The domain architecture associated with each ACP clade is shown. A linear schematic of ACP domain secondary structure is displayed with positions of residues involved in the PksJ KR2:ACP4 interaction highlighted. The residues at the equivalent positions on PksJ ACP3a are shown for comparison. Residues Q51 and E25 are highly conserved within ‘clade k’ ACP domains, and have been shown to be important contributors to the PksJ ACP4:KR2 interface. The equivalent positions in ‘clade h’ ACP domains are poorly conserved, and often with different chemical functionality. The phosphopantetheine-attachment site (ACP4: S46 and ACP3a: S62) is highlighted with an asterisk. (B) Mass spectra of ketoreduction assays monitored by 4′-phosphopantetheine ejection showing productive interactions between cognate ACP:KR pairs, but no ketoreduction between non-cognate pairs. (C) Diagrammatic representations of PksJ ACP4 domain (grey), PksJ ACP3a domain (black) and contributions of an associated mutants and chimera. Relative ketoreduction activities of the PksJ ACP3a (V67Q) mutant, PksJ ACP3a (I41E, V67Q) double mutant and PksJ ACP4 [H_I_–H_II_]/ACP3a [H_III_–H_IV_] chimera with PksJ KR2 are shown, and the values are expressed as a percentage of WT PksJ KR2 activity. Errors are ±standard deviation from the mean, where *n* = 3 and n.d. = not detected.

Having identified the molecular details of the PksJ KR2:ACP4 interface, we directed our attention to understanding the specificity of this interaction with respect to *trans*-AT PKS module architectures. At the phylogenetic level, PksJ ACP4 belongs to a discrete clade of ACP domains that conform to the KR^A^–
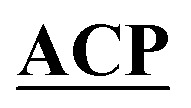
–KS^0^//DH–ACP–KS domain architecture (clade k, ESI Fig. S11†), which appear to have co-evolved together as a functional set of domains, or migratory unit (MU), responsible for installation of a *cis*-configured double bond.^43,44^ In comparison, the preceding module of the bacillaene PKS generates a fully saturated intermediate *via* the sequential activity of a KR^B^ domain, DH domain and a *trans*-acting ER domain (Fig. 1A).^45^ The module contains two identical copies of the ACP domain positioned in tandem; a common occurrence which coincides with recruitment of *trans*-acting domains to the assembly line.^11^ The ACP domains from this module (PksJ ACP3a and ACP3b) both sit in a different phylogenetic clade to that of PksJ ACP4, reflective of the MU from which it originates (DH–KR^B^–
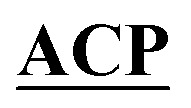
–KS + ER) (clade h, ESI Fig. S11†). At the sequence level, ACP domains from ‘clade k’ (*e.g.* PksJ ACP4) and ‘clade h’ (*e.g.* PksJ ACP3a/b) are distinct from each other, with sequence logos of these clades highlighting the differences in conserved residues (Fig. 5A). Interestingly, many of the residues found to be important in the PksJ ACP4:KR2 domain interaction are also highly conserved within the sequences of ‘clade k’ ACP domains, in particular: E25, I47 and Q51. Residues at the equivalent positions of ‘clade h’ ACP domains are poorly conserved and often of different functionality, with the notable exception of the position corresponding to I47 (Fig. 5A). Furthermore, when considering the PksJ KR2 domain, it is worth noting that the position of R228 in the loop region involved in NADPH-binding is always occupied by a Gly residue in *cis*-AT PKS KR domains.^31,46^ Analysis of KR domain sequences from *trans*-AT PKSs reveals that Gly is also conserved at this position in the overwhelming majority of cases, except for the KR domains that are part of ‘clade k’ migratory units, which have Arg highly conserved (ESI Fig. S12†). These observations led us to postulate that ACP:KR interactions in *trans*-AT PKSs are highly optimised within their respective MUs and are unable to interact in non-cognate pairings.

In order to test this hypothesis, excised PksJ ACP3a and PksJ KR1 domains (*i.e.* representatives of ‘clade h’) were cloned and overproduced in *E. coli* followed by intact protein MS analysis to confirm their identity (Fig. 1A and ESI Fig. S1†). Using the ketoreduction assay, incubation of 3-keto-butyryl-PksJ ACP3a domain with the PksJ KR1 domain resulted in complete conversion to the 3-hydroxy-butyryl-PksJ ACP3a species after 10 min. However, the equivalent reaction using the PksJ KR2 domain resulted in no ketoreduction product, suggesting that PksJ ACP3a domain is unable to interact with the PksJ KR2 domain (Fig. 5B). This incompatibility is likely due to PksJ ACP3a domain possessing none of the residue functionality at key positions required to facilitate interaction with the PksJ KR2 domain. Accordingly, the pairing of PksJ ACP4 and PksJ KR1 also resulted in no ketoreduction activity (Fig. 5B), implying that a distinct binding epitope exists on the PksJ ACP3a domain to facilitate this interaction. These observations suggest that ACP and KR domains from *trans*-AT PKSs are only capable of interacting within their module/MU, which have co-evolved a highly optimised protein–protein interface. This is in stark contrast to *cis*-AT PKSs, where ACP and KR domains appear to be unable discriminate between cognate and non-cognate pairings,^29,30^ and can be explained by the different evolutionary origins of *cis*- and *trans*-AT PKS systems.^43,44,47^ Observations from the PksJ ACP4:KR2 interface imply that the residues on the ACP domain of the PksJ ACP3a:KR1 interface are likely to be highly conserved. Although the equivalent positions of the KR-binding epitope from the PksJ ACP4 domain do not correlate with conserved residues on the PksJ ACP3a domain, residues in adjacent positions are highly conserved (T66, N70 and N73) (Fig. 5A). We postulate that these residues may serve similar roles to that of Q51, R55 and R58 in the PksJ ACP4 domain, with the different functionality and positioning contributing to the interaction specificity. Furthermore, a plausible docked model of the PksJ ACP3a:KR1 complex can be obtained assuming an analogous binding mode as described above, which position the PksJ ACP3a domain in a near identical position to that of the PksJ ACP4:KR2 complex with the highly conserved residues contributing to the interface (ESI Fig. S13†). This suggests that, whilst the general region of the interface is the same, subtle variations in residues on both the ACP and KR domains provide the specificity.

Although the PksJ ACP3a domain was unable to interact productively with the PksJ KR2 domain, residues required for the binding epitope on the ACP domain had been elucidated from our mutagenesis data and docked model. Using this knowledge, a set of mutations and chimeric constructs were produced to engineer the PksJ ACP3a domain towards a productive interaction with the PksJ KR2 domain. In the first instance, a hepta-mutant construct of was produced, where all seven residues of the PksJ KR2-binding epitope were introduced into the equivalent positions on the PksJ ACP3a domain. Overexpression of the hepta-mutant construct in *E. coli* yielded insoluble protein, as did a chimeric construct replacing H_II_ of the PksJ ACP3a domain with that of the PksJ ACP4 domain. The mutagenesis data described previously indicated that Q51 plays critical role in the interaction epitope (Fig. 2D and E) and is highly conserved in ‘clade k’ ACP domains, while the equivalent position on the PksJ ACP3a domain is valine (V67) (Fig. 5A). Although a PksJ ACP3a(V67Q) construct yielded soluble recombinant protein, this single mutation did not promote interaction with the PksJ KR2 domain in the ketoreduction assay (Fig. 5C). The E25 residue in the PksJ ACP4 domain is equally conserved, however introduction of a I41E mutation to create a PksJ ACP3a (I41E, V67Q) double-mutant was also unable to stimulate interaction with the PksJ KR2 domain (Fig. 5C). These findings are congruent with observations from the docked model that suggest the role of Q51 is to position other residues, but notably not E25, on the ACP domain in the correct orientation for interaction with the KR domain (ESI Fig. S10D†). Although E25 appears to interact with R228 on the PksJ KR2 domain, with an intra-molecular orientation effect from R27 (ESI Fig. S10C†), introduction of this residue into a non-cognate ACP domain is unlikely to drive complex formation.

Surprisingly, a chimeric construct comprised of H_I_–H_II_ from the PksJ ACP4 domain, and H_III_–H_IV_ from the PksJ ACP3a domain yielded soluble recombinant protein, which encompassed all seven residues of the PksJ KR2 domain interaction epitope. Furthermore, the PksJ ACP4 [H_I_–H_II_]/ACP3a [H_III_–H_IV_] chimera was able to restore ketoreduction activity to ∼60% of WT PksJ ACP4 domain levels (Fig. 5C). Despite harbouring all critical residues, only partial restoration of activity may be the result of sub-optimal packing of the chimeric 4α-helix bundle, distorting formation of the interaction epitope on the ACP domain surface. However, these observations suggest that ACP domains can be engineered to interact with non-cognate partners, providing the binding epitope is adequately preserved.

## Conclusions

In summary, we have elucidated molecular details of the interaction interface between an ACP domain and a KR domain from a *trans*-AT PKS, allowing production of a docked model driven by residue-level experimental data. The KR-binding epitope of the ACP domain encompasses key residues situated on H_I_ and H_II_, and combined with previous observations,^17^ the ACP domain docks at the same KR_C_ subdomain site in both *cis*- and *trans*-AT systems. However, unlike *cis*-AT PKSs,^29,30^ we have shown that ACP domains from *trans*-AT PKSs are highly specific for the KR domain within their module/migratory unit; a result of the distinct evolutionary origins of *cis*- and *trans*-AT PKSs. Exchange of the H_I_–H_II_ region within an ACP domain promoted communication with a non-cognate KR domain, highlighting the minimum requirements for interaction with the KR domain and the potential for manipulation of the ACP domain. Taken together, these results provide important domain-level compatibility rules for engineering efforts on *trans*-AT PKS pathways, and serves to highlight the requirement for a fundamental understanding of the carrier protein interaction network to ensure these endeavours are successful.

## Data availability

The 200 ns MD trajectory stripped of water and ions with frames every 1 ns is available for download from Mendeley Data DOI: 10.17632/8shpf4mrs6.1.

## Author contributions

M. J. conceived and designed the study. M. P. and M. J. generated expression constructs, overproduced and purified proteins, constructed mutant plasmids and performed biochemical assays. M. J. performed and analysed the carbene footprinting experiments. A. G., and J. R. L. conducted docking studies and molecular dynamics simulations. M. J. and M. P. wrote the manuscript with input from all authors.

## Conflicts of interest

There are no conflicts to declare.

## Supplementary Material

SC-012-D1SC03478B-s001
