# Supplementary material for: Molecular basis for acyl carrier protein–ketoreductase interaction in trans-acyltransferase polyketide synthases
Source: Chem Sci. 2021 Sep 30;12(41):13676–85. doi: 10.1039/d1sc03478b (PMC8549798; doi:10.1039/d1sc03478b)
Supplement: SC-012-D1SC03478B-s001 [file SC-012-D1SC03478B-s001.pdf]

Electronic Supplementary Material (ESI) for Chemical Science.
This journal is © The Royal Society of Chemistry 2021

Supporting Information

Molecular basis for acyl carrier protein-ketoreductase interaction in *trans*-acyltransferase polyketide synthases.

Munro Passmore[a], Angelo Gallo[a], Józef R. Lewandowski[a] and Matthew Jenner[a,b]*

[a] Department of Chemistry, University of Warwick, Coventry, CV4 7AL, UK.
[b] Warwick Integrative Synthetic Biology Centre, University of Warwick, Coventry CV4 7AL, UK.

* Corresponding author: Matthew Jenner (m.jenner@warwick.ac.uk).

1. Supplementary Figures

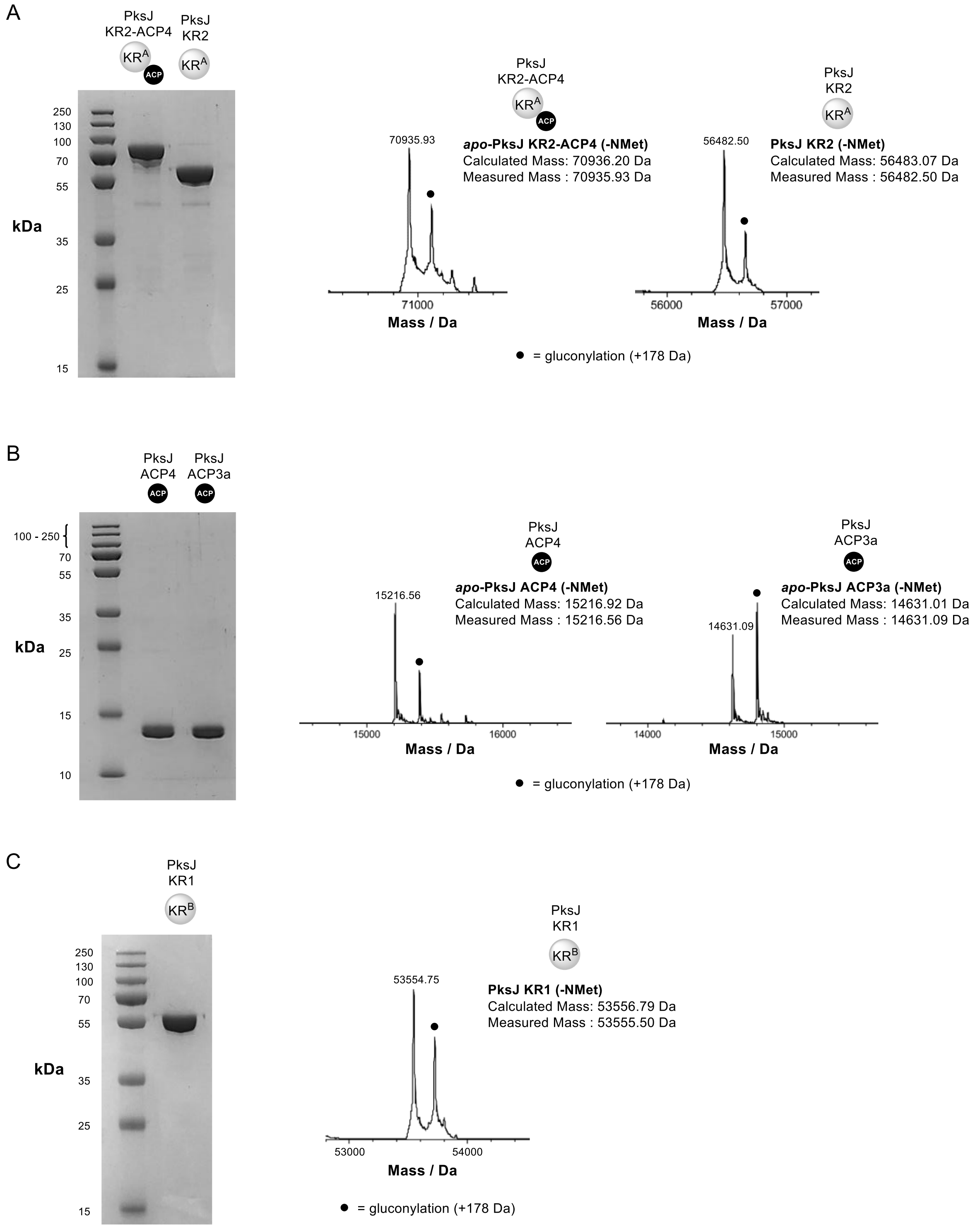

Figure S1: SDS-PAGE and mass spectrometry analysis of wild-type purified recombinant proteins (**A**). 10 % SDS-PAGE gel containing (from left to right): PksJ KR2-ACP4 didomain (71 kDa), PksJ KR2 domain (56 kDa). Deconvoluted mass spectra of each (di)-domain is shown next to the gel, with calculated and observed masses detailed. (**B**). 12 % SDS-PAGE gel containing (from left to right): PksJ ACP4 domain (15 kDa), PksJ ACP3a domain (14 kDa). (**C**). 10 % SDS-PAGE gel containing PksJ KR1 domain (53 kDa). Deconvoluted mass spectra of each domain is shown next to the gel, with calculated and observed masses detailed. The black dot indicates the typical post-translational gluconylation of the His-Tag from pET28a(+) recombinant proteins.[1]

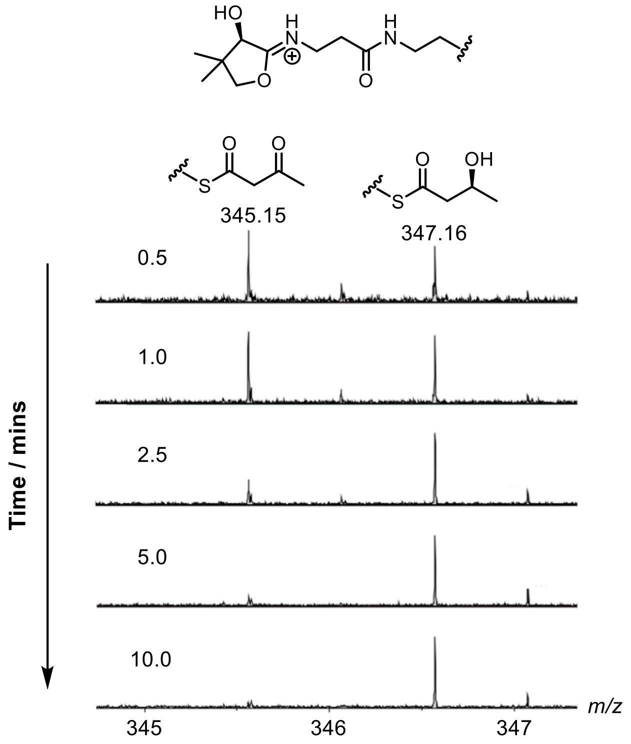

Figure S2: Time-course analysis of conversion of AcAc-ACP4 to β-OH-ACP4 catalyzed by PksJ KR2. Phosphopantetheine ejection spectra of the reaction between PksJ KR2 and AcAc-PksJ ACP4 over a 10 min time-course. The ratio between 3-keto-butyryl-Pant and (3*S*)-3-hydroxy-butyryl-Pant was measured for each time point. The reaction is nearing completion at the 5 min time point, and this was taken as the length of time to run subsequent assays with the mutant PksJ ACP4 domains.

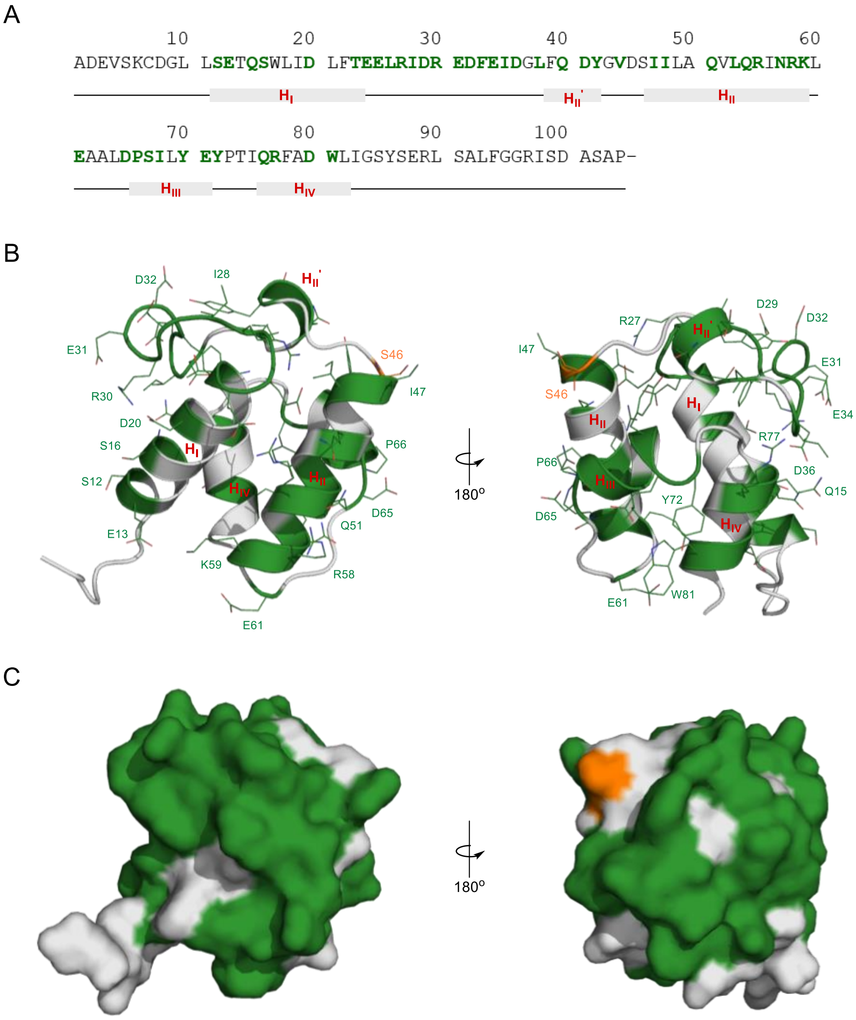

Figure S3: Sequence and surface coverage of PksJ ACP4 by alanine scanning mutagenesis.
(**A**). The amino acid sequence of the PksJ ACP4 construct used in this study is shown (minus His-Tag region), with predicted secondary structure assignments below. Residues highlighted in blue were mutated to alanine and resulted in soluble protein. (**B**). Homology model of PksJ ACP4 shown in cartoon representation. Residues that were mutated to alanine are highlighted in blue and shown in line format. (**C**). Surface representation of PksJ ACP4 homology model. Blue regions correlate to residues mutated to alanine in the cartoon representation. Approximately 95 % of the solvent exposed non-Ala/Gly residues from the core 4-helix bundle were covered by the scanning alanine mutagenesis approach (excluding Ser46).

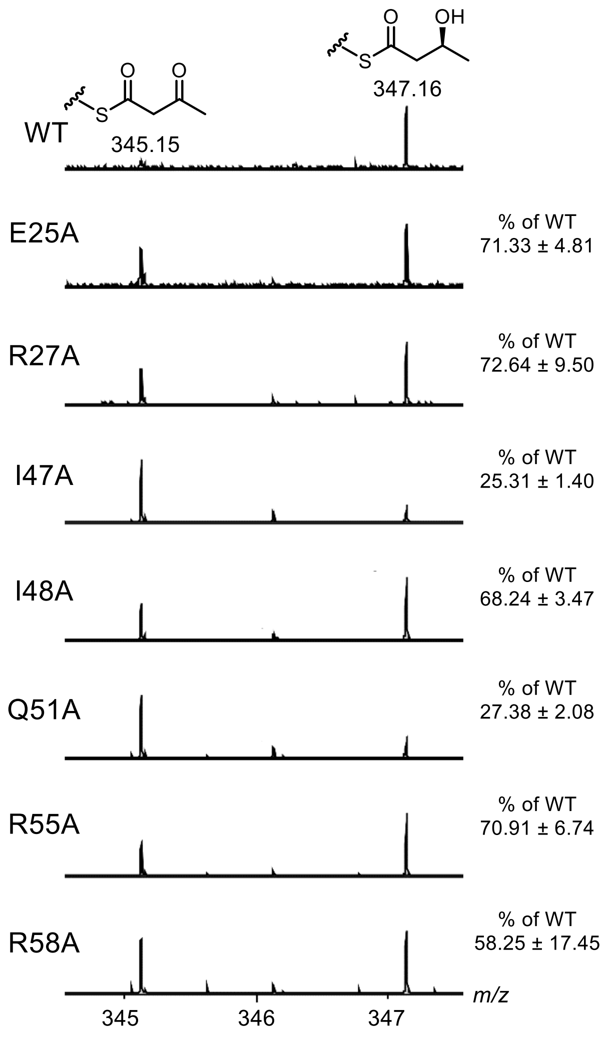

Figure S4: Ppant ejection spectra of PksJ ACP4 alanine mutants identified at the ACP:KR binding epitope. Mass spectra of ketoreduction assays monitored by Ppant ejection for PksJ ACP4 alanine mutants with ketoreduction activity < 80 % with PksJ KR2. Values are reported relative to WT PksJ ACP4 activity and errors represent ± standard deviation from the mean, where $n = 3$.

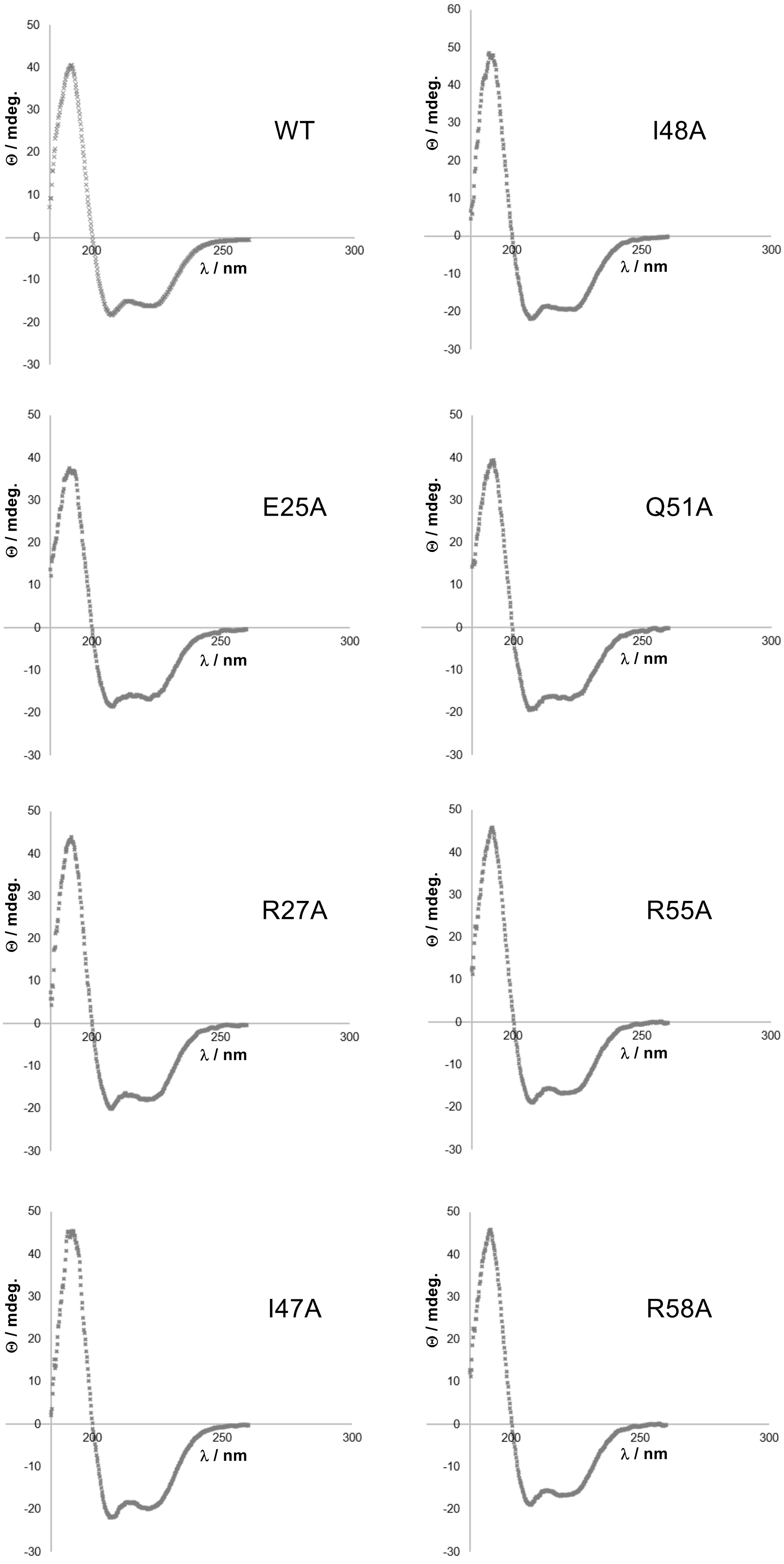

Figure S5: Circular dichroism spectroscopic analysis of PksJ ACP4 mutants. Overlaid CD spectra for WT PksJ ACP4 and all alanine mutants which disrupted the interaction with PksJ KR2. The CD spectra obtained for each mutant are in agreement with the WT PksJ ACP4 domain, suggesting no loss/gain of secondary structure has occurred upon mutation.

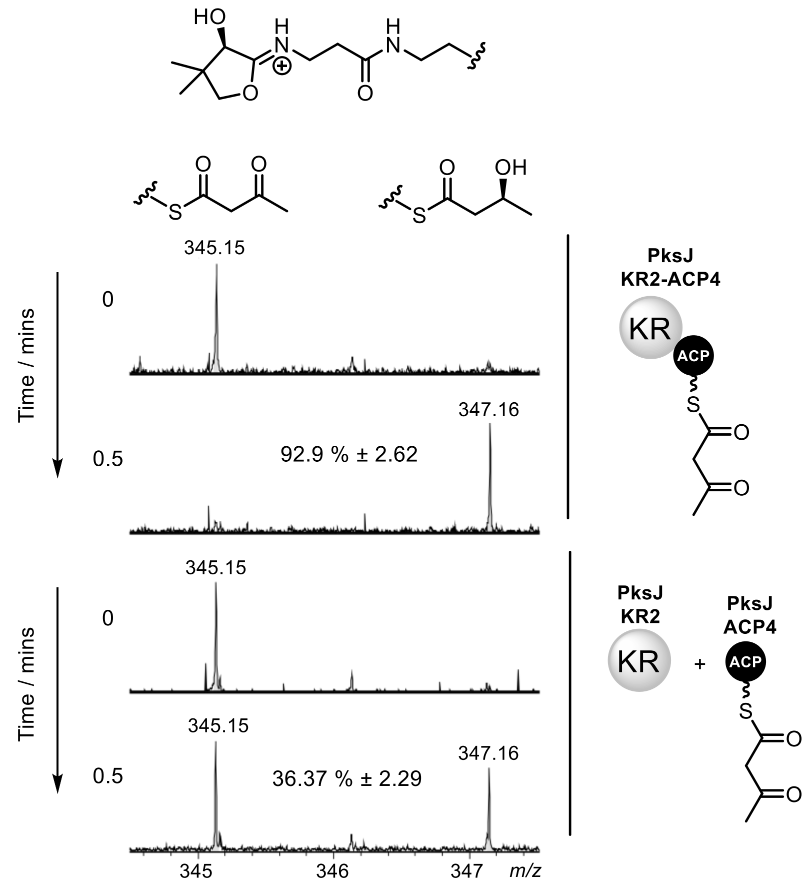

Figure S6: Comparison of ketoreduction activity between PksJ KR2-ACP4 and PksJ KR2 + PksJ ACP4. Mass spectra of ketoreduction assays monitored by Ppant ejection for PksJ KR2-ACP4 (top) and PksJ KR2 + PksJ ACP4 (bottom) after 0.5 min incubation. Percentage conversion form 3-keto-butyryl-Pant and (3*S*)-3-hydroxy-butyryl-Pant is displayed, and errors represent ± standard deviation from the mean, where *n* = 3.

PksJ KR2-ACP4 Amino Acid Sequence:

```
        10         20         30         40         50         60
SERDKKELVN AIEDRAACFL TKQWSLSPIG SAVPGTRTVA ILCCQETADL AAEVSSYFPN

        70         80         90        100        110        120
HLLIDVSRIE NDQSDIDWKE FDGLVDVIGC GWDDEGRLDW IEWVQRLVEF GHKEGLRLLC

       130        140        150        160        170        180
VTKGLESFQN TSVRMAGASR AGLYRMLQCE YSHLISRHMD AEEVTDHRRL AKLIADEFYS

       190        200        210        220        230        240
DSYDAEVCYR DGLRYQAFLK AHPETGKATE QSAVFPKDHV LLITGGTRGI GLLCARHFAE

       250        260        270        280        290        300
CYGVKKLVLT GREQLPPREE WARFKTSNTS LAEKIQAVRE LEAKGVQVEM LSLTLSDDAQ

       310        320        330        340        350        360
VEQTLQHIKR TLGPIGGVIH CAGLTDMDTL AFIRKTSDDI QRVLEPKVSG LTTLYRHVCN

       370        380        390        400        410        420
EPLQFFVLFS SVSAIIPELS AGQADYAMAN SYMDYFAEAH QKHAPIISVQ WPNWKETGMG

       430        440        450        460        470        480
EVTNQAYRDS GLLSITNSEG LRFLDQIVSK KFGPVVLPAM ANQTNWEPEL LMKRRKPHEG

       490        500        510        520        530        540
GLQEAALQSP PARDIEEADE VSKCDGLLSE TQSWLIDLFT EELRIDREDF EIDGLFQDYG

       550        560        570        580        590        600
VDSIILAQVL QRINRKLEAA LDPSILYEYP TIQRFADWLI GSYSERLSAL FGGRISDASA

P-
```

Sequence coverage of PksJ KR2 domain (1 – 473) = **67.4 %**

Figure S7: Sequence coverage of PksJ KR2 domain from carbene footprinting experiments from tryptic digests. Masked regions are highlighted in red, and unmasked regions are highlighted in blue. Peptides that were detected but with unchanged levels of labelling are highlighted in wheat. The 24-residue linker between the KR and ACP domains is highlighted in green, and the sequence corresponding to the PksJ ACP4 domain is highlighted in grey. Sequence coverage is calculated for the PksJ KR2 domain (residues 1 – 473) only.

A

B

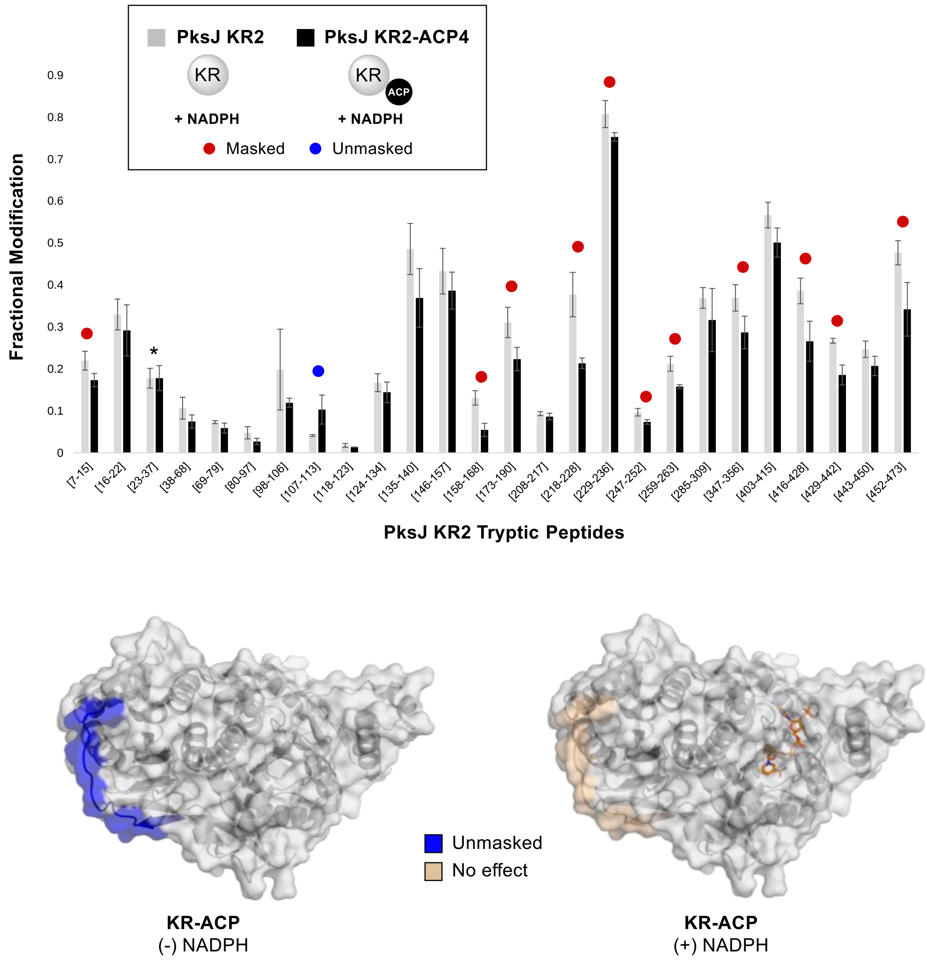

Figure S8: Carbene footprinting of PksJ KR-ACP in the presence of NADPH.
A). Fractional modification of PksJ KR2 tryptic peptides arising from photochemical labelling in the absence (grey bars) and presence (black bars) of PksJ ACP4 domain (covalently tethered via PksJ KR2-ACP4 didomain construct). Error bars represent ± standard deviation from the mean, where *n* = 3. Significant differences (Student's t-test, P < 0.05) are highlighted with a red (masked) or blue (unmasked) dot. The peptide [Q23-37] that differs in its differential modification when compared to footprinting in the absence of NADPH is indicated by an asterisk. **B**). Structure of PksJ KR2 (from PDB: 5KTK) showing the location the [Q23-R37] peptide. The peptide is unmasked upon binding of PksJ ACP4 in the absence of NADPH (left). In the presence of NADPH, binding of PksJ ACP4 does not induce unmasking in this region, suggesting some stablization of the structure whilst NADPH is bound.

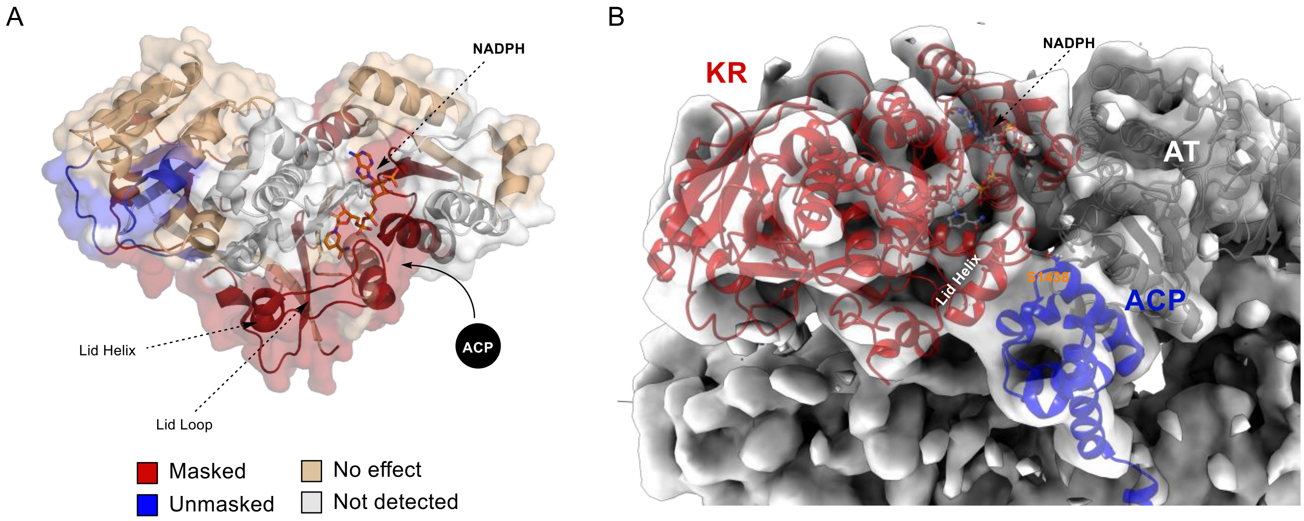

Figure S9: Similar ACP-binding sites on KR domains from *cis*- and *trans*-AT PKSs.
(**A**). Structure of PksJ KR2 (from PDB: 5KTK)[2] showing the locations of masked (red), unmasked (blue) and unaffected (wheat) peptide regions in the presence of PksJ ACP4 domain. The experimentally determined ACP docking site is indicated. (**B**). Cryo-EM map of PikAIII[3] with KR (DEBS M1, PDB: 2FR0)[4], AT (DEBS M5, PDB: 2HG4)[5] and ACP (DEBS M2, PDB: 2JU2)[6] domains fitted in ChimeraX[12]. The KR and ACP domains are highlighted in red and blue, respectively. The location of the Ppant modified Ser1436 residue on the ACP domain is highlighted and shown in stick representation.

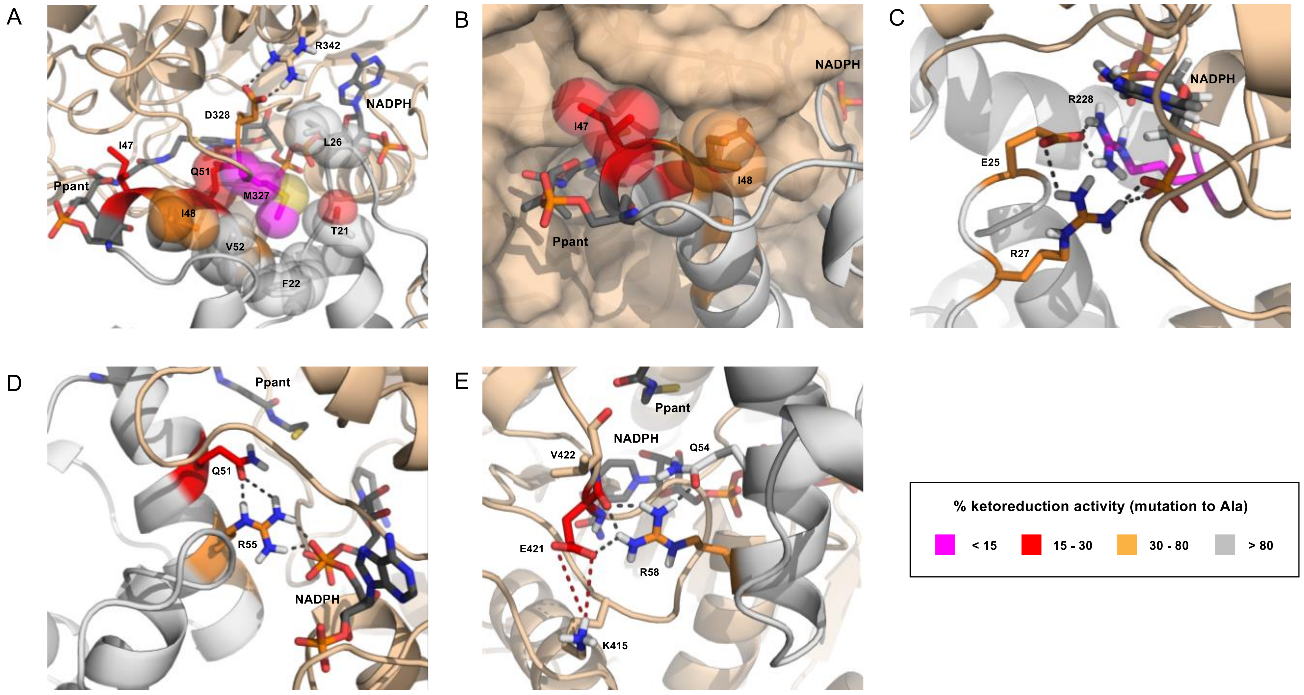

Figure S10: Perspectives of the docked PksJ KR2:ACP4 complex showing the interactions of key residues. Relevant residues are shown as sticks and/or spheres and coloured according to the ketoreduction activity profile displayed in the inset. (**A**). A set of hydrophobic residues on PksJ ACP4 domain (F22, L26, I48, V52) shown as spheres form a pocket around M327 on the PksJ KR2 domain surface. In order for M327 to enter the hydrophobic groove of the ACP domain, to allow M327 to interface with the hydrophobic groove D328 is pulled out of spatial proximity by R342. (**B**). Residues I47 and I48 on PksJ ACP4 domain plug a hydrophobic pocket on the PksJ KR2 domain surface. The orientation of I47 guides the Ppant arm into the KR domain active site. (**C**). Contacts between E25 on the PksJ ACP4 domain and R228 on the PksJ KR2 domain are shown. The orientation of E25 is stipulated by and intra-molecular interaction with R27, which also appears to make charged contacts with NADPH. (**D**). Interaction network between Q51 and R55 on the PksJ ACP4 domain. The Q51 residue dictates the orientation of R55, which in turn forms an interaction with NADPH. (**E**). The R58 residue on the PksJ ACP4 domain interacts with the side chain and the backbone carbonyl of E421 on the PksJ KR2 domain. An intra-molecular interaction between R58 and Q54 stabilises the positioning of the R58 side chain. During the time-course of the simulation, the E421 side chain oscillates between interactions with R58 and K415. The E421:K415 interaction stabilises a loop region which provides a platform for the PksJ ACP4 domain to dock correctly.

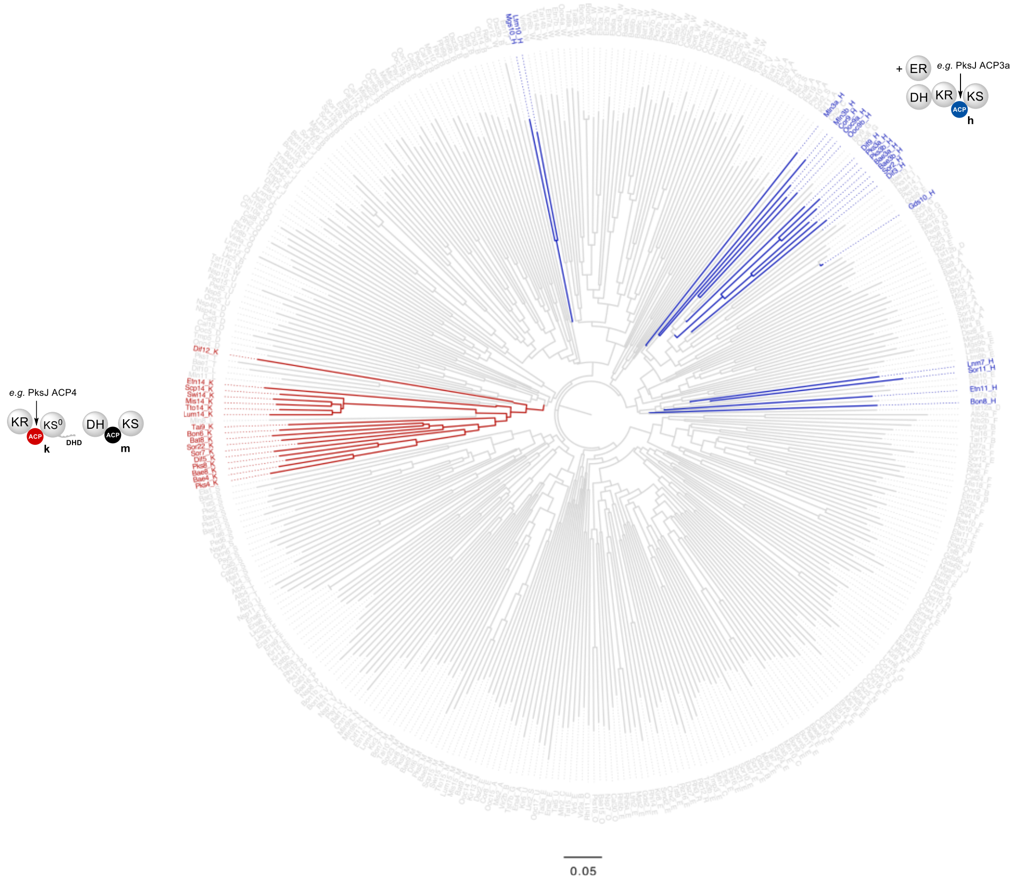

Figure S11: Phylogenetic analysis of ACP domains from *trans*-AT PKSs. Cladogram comprised of ACP domains from *trans*-AT PKS pathways, constructed using previously reported methods and sequence sets.[7] The phylogenetic clades that encompass PksJ ACP4 (clade k) and PksJ ACP3a (clade h) are highlighted in red and blue, respectively, highlighting their evolutionary differences. The migratory unit (MU) architecture for these clades is displayed.

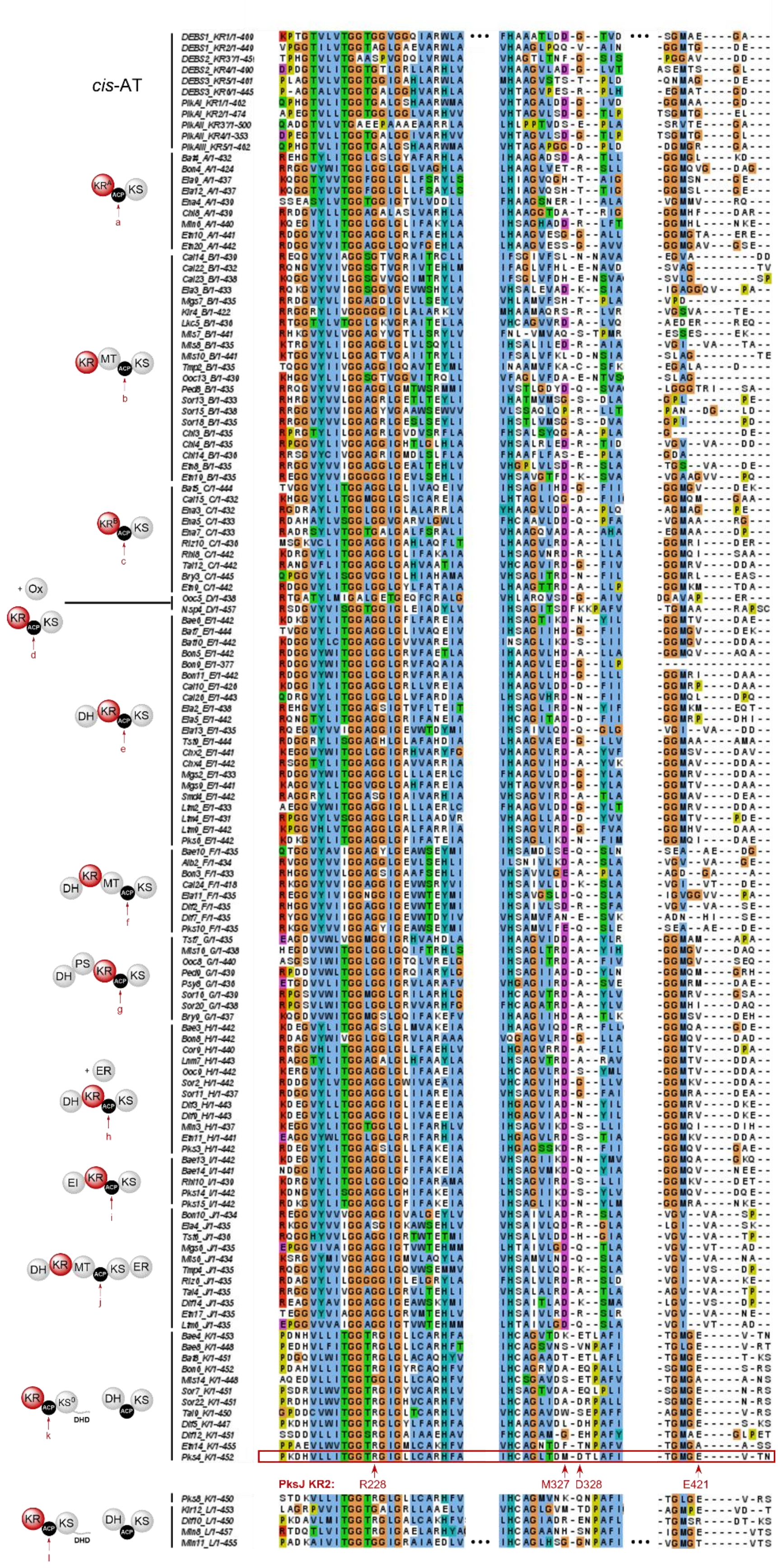

Figure S12: Phylogenetic analysis of ACP domains from *trans*-AT PKSs. Cladogram comprised of ACP domains from *trans*-AT PKS pathways, constructed using previously reported methods and sequence sets.[7] The phylogenetic clades that encompass PksJ ACP4 (clade k) and PksJ ACP3a (clade h) are highlighted in red and blue, respectively, highlighting their evolutionary differences. The migratory unit (MU) architecture for these clades is displayed.

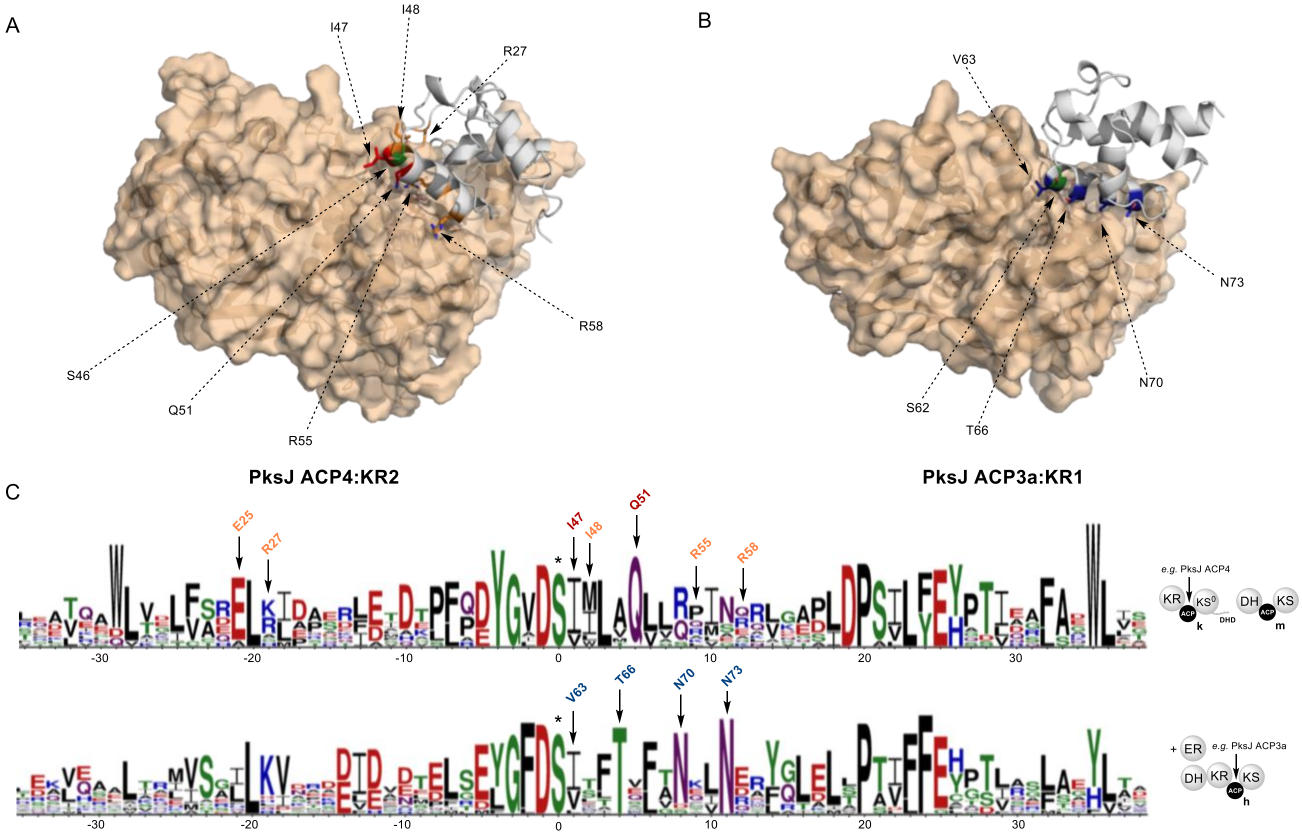

Figure S13: Docked models of cognate ACP:KR domain complexes. (**A**). Docked model of the PksJ ACP4:KR2 complex. The PksJ ACP4 domain residues shown to be involved in the interface are shown as sticks and coloured according to effect on relative ketoreduction activity. (**B**). Docked model of the PksJ ACP3a:KR1 complex using the crystal structure of PksJ KR1 domain (PDB: 4J1Q) and a homology model of the PksJ ACP3a domain. Highly conserved residues within 'clade h' ACP domains are highlighted in blue, and appear to contribute to the interface in a similar way to the PksJ ACP4:KR2 complex. (**C**). Sequence logos for *trans*-AT PKS ACP domains from the phylogenetic clades: 'clade k', (*top,* e.g. PksJ ACP4) and 'clade h' (*bottom*, e.g. PksJ ACP3a). Residues highlighted on the structures in panels (**A**) and (**B**) are indicated by arrows on the sequence logos.

2. Biochemical, Biophysical and Computational Methods

2.1 Cloning and Site Directed Mutagenesis

The amplification of PksJ ACP4, PksJ ACP3a, PksJ KR1 and PksJ KR-ACP2 was performed from *B. subtilis* strain 168 gDNA using Q5 DNA polymerase (NEB) and the primers detailed in **Table 1**. PCR products were separated on a 1 % agarose gel and bands were excised and purified with a GeneJet gel extraction kit (Thermo Scientific). The purified inserts were digested using BamHI and XhoI, and subsequently ligated with pET28a(+) using T4 ligase (NEB) which had been pre-digested with the corresponding restriction enzymes. In the case of PksJ ACP3a, the PCR product was ligated into a modified version of pET28a where two additional His residues are introduced into the His-Tag region (see amino acid sequence in **Section 3**). The ligation mixture was used to transform *E. coli* TOP10 cells (Invitrogen), which were plated on LB agar containing kanamycin (50 µg/mL). Colonies were picked and grown overnight in LB media containing (50 µg/mL). Plasmids were isolated from cultures using a miniprep kit (Thermo), and the inserts were sequenced to verify their integrity.

The PksJ KR2 domain expression construct was produced using the Q5 site-directed mutagenesis kit (NEB), using the pET28a_PksJ_KR2-ACP4 construct as a template. The resulting PCR product incorporated a STOP codon between the KR and ACP domains, and was processed according to the manufactures protocol to generate the pET28a_PksJ_KR2 construct, which was sequenced to verify the correct mutation and plasmid integrity. All PksJ ACP4, PksJ ACP3a and PksJ KR2 alanine mutants were constructed using the Q5 site-directed mutagenesis kit (NEB), using primers detailed in **Tables 2** and **3** introducing a (XXX→GTC, X→Ala) mutation in each case. PCR products were processed according to the manufactures protocol, and resulting plasmids sequenced to verify correct mutation. The PksJ ACP4 [H_I-H_{II}] / ACP3a [H_{III}-H_{IV}] chimera and PksJ ACP3a (I41E, K43R, V63I, T64I, V67Q, K71R, E74R) hepta-mutant constructs were gene synthesised (Epoch Life Sciences) and subcloned into pET24a with a custom $pHis_8$ tag.

2.2 Protein Overproduction and Purification

A single colony of *E. coli* BL21(DE3) that had been transformed with the appropriate expression vector was picked and used to inoculate LB medium (5 or 10 mL) containing kanamycin (50 µg/mL). The resulting culture was incubated overnight at 37˚C and 180 rpm then used to inoculate LB medium (0.5 or 1 L) containing kanamycin (50 µg/mL). The resulting culture was incubated at 37˚C and 180 rpm until the optical density of the culture at 595 nm reached 0.6, then IPTG (1 mM) was added and growth was continued overnight at 15˚C and 180 rpm. The cells were harvested by centrifugation (4,000 x g, 15 min, 4˚C) and re-suspended in buffer (20 mM Tris-HCl, 100 mM NaCl, 20 mM Imidazole, pH 7.4) at 10 mL/L of growth medium then lysed using a Constant Systems cell disruptor.

The lysate was centrifuged (37,000 x g, 30 min, 4˚C) and the resulting supernatant was loaded onto a HiTrap FF Chelating Column (GE Healthcare), which had been pre-loaded with 100 mM $NiSO_4$ and equilibrated in re-suspension buffer (20 mM Tris-HCl, 100 mM NaCl, 20 mM Imidazole, pH 7.4). Proteins were eluted in a stepwise manner using re-suspension buffer containing increasing concentrations of imidazole – 50 mM (5 mL), 100 mM (3 mL), 200 mM (3 mL) and 300 mM (3 mL). The presence of the protein of interest in fractions was confirmed by SDS-PAGE, and an additional gel filtration step (Superdex 75/200, GE Healthcare) was used to further purify proteins where necessary. Fractions containing the protein of interest were pooled and concentrated to 250-400 µM using a Viva-Spin centrifugal concentrator (Sartorius) at an appropriate MWCO (10 kDa for PksJ ACP4, PksJ ACP3a and PksJ KR2; 30 kDa for PksJ KR2-ACP4). Samples were snap-frozen in liquid N_2 and stored at -80˚C.

2.3 Ketoreduction Assays

PksJ ACP4 or associated alanine mutants (200 µM) were converted to their acetoacetyl (AcAc)-form by incubation in 20 mM Tris, 100 mM NaCl, 2 µM Sfp PPtase, 1 mM AcAc-CoA and 10 mM $MgCl_2$ in a total volume of 50 µL for 30 min at 25 °C. Ketoreduction assays were performed in 30 µL total volume of 20 mM Tris, 100 mM NaCl. AcAc-ACP domains (50 µM) were incubated with PksJ KR2 (or associated alanine mutants) (50 µM) and NADPH (500 µM) for 5 min at RT, followed by quenching by addition of formic acid to a final concentration of 1% v/v. Samples were then diluted 5-fold with mQH_2O, then analysed by UHPLC-ESI-Q-TOF-MS.

2.4 UHPLC-ESI-Q-TOF-MS Analysis of Intact Proteins and Phosphopantetheine Ejection

All ketoreduction assays were analysed on a Bruker MaXis II ESI-Q-TOF-MS connected to a Dionex 3000 RS UHPLC fitted with an ACE C_4-300 RP column (100 x 2.1 mm, 5 µm, 30 °C). The column was eluted with a linear gradient of 5 – 100% MeCN containing 0.1% formic acid over 30 min. The mass spectrometer was operated in positive ion mode with a scan range of 200 – 3000 *m/z*. Source conditions were: end plate offset at −500 V; capillary at −4500 V; nebulizer gas (N_2) at 1.8 bar; dry gas (N_2) at 9.0 L min^{-1}; dry temperature at 200 °C. Ion transfer conditions were: ion funnel RF at 400 Vpp; multiple RF at 200 Vpp; quadrupole low mass at 200 *m/z*; collision RF at 2000 Vpp; transfer time at 110.0 µs; pre-pulse storage time at 10.0 µs. Measured masses for the intact *apo*-, *holo*- and AcAc-ACP4 species are displayed in **Table 4**. Collision-induced dissociation (collision energy fixed at 8 eV) applied to all charge state species promoted phosphopantetheine ejection ions.

2.5 Carbene Footprinting: Photolysis and Data Analysis

A 50 µM solution of PksJ KR2 or *apo*-PksJ KR2-ACP4 in 20 µL of 20 mM Tris, 100 mM NaCl (+/- 100 µM NADPH) was mixed with an equal volume of a 10 mM solution of aryldiazirine photoreagent in the same buffer. The mixture was left to equilibrate for 5 minutes at room temperature before 6 µL aliquots were placed in crystal clear vials (Fisher Scientific UK) and snap-frozen in liquid nitrogen. The labelling reaction was initiated by irradiating the mixture using the output of an optical parametric amplifier (TOPAS, light conversion) at a wavelength of 350 nm with a repetition rate of 1 kHz, an energy per pulse of 10 mJ, and a pulse duration of 40 fs. The TOPAS was pumped by the 800 nm fundamental output of a commercially available Ti:sapphire ultrafast laser system (Spitfire Ace PA Pro, Newport-Spectra Physics) at 2.5 W average power, 1 kHz repetition rate, and 40 fs pulse duration. The frozen samples were irradiated for 16 s. All experiments were performed in triplicate. Following irradiation, samples were thawed, reduced (10 mM DTT, in 10 mM ammonium bicarbonate), alkylated (55 mM Iodoacetamide, in 10 mM ammonium bicarbonate) and incubated at 37 °C with trypsin overnight (1:20 protease/protein ratio in 10 mM ammonium bicarbonate).

The analysis of the digests was carried out on a Bruker MaXis II ESI-Q-TOF-MS connected to a Dionex 3000 RS UHPLC fitted with an ACE C_{18} RP column (100 x 2.1 mm, 5 µm, 30 °C). The column was eluted with a linear gradient of 5 – 100 % MeCN containing 0.1% formic acid over 40 min. The mass spectrometer was operated in positive ion mode with a scan range of 200 – 3000 *m/z*. Source conditions were: end plate offset at −500 V; capillary at −4500 V; nebulizer gas (N_2) at 1.6 bar; dry gas (N_2) at 8.0 L min^{-1}; dry temperature at 180 °C. Ion transfer conditions were: ion funnel RF at 200 Vpp; multiple RF at 200 Vpp; quadrupole low mass at 55 *m/z*; collision energy at 5.0 eV; collision RF at 600 Vpp; ion cooler RF at 50–350 Vpp; transfer time at 121 s; pre-pulse storage time at 1 µs. A previously described method was used to quantitate the fraction of each peptide modified.[8] Briefly, the chromatograms for each singly-labelled and unlabelled peptide were extracted within a range of ± 0.02 *m/z* and the spectrum for each peak was manually inspected to ensure the sampling of the correct ion only. The peptide fractional modification was calculated using **Equation 1**.

Equation 1:

$$P = \frac{A_{labelled}}{A_{labelled} + A_{unlabelled}}$$

Where $A_{labelled}$ and $A_{unlabelled}$ correspond respectively to the peak area of each labelled and unlabelled peptide. Differences in the extent of labelling between peptides were considered significant when the *P* value obtained from a Student t-test was < 0.05.

2.6 Circular Dichroism Analyses of ACP Domains

Circular dichroism spectra of PksJ ACP4 and associated mutants were acquired on a JASCO J-1500 spectropolarimeter, using a 1 mm path length quartz cuvette. Spectra were acquired between 190 and 260 nm at room temperature. Before analysis protein samples were exchanged into CD buffer (0.5 mM Tris HCl, 5 mM NaCl, pH 7.4) and adjusted to a final concentration of 0.1 mg/mL.

2.7 Homology Modelling, Residue-Guided Protein Docking and Molecular Dynamics

2.7.1 Homology Modelling

Homology modelling of PksJ ACP4 domain was conducted using the I-TASSER webserver[9], and the *apo*-MmpA ACP-ACP di-domain structure as a template (PDB: 2L22).[10] The structure was then further refined using the MolProbity server.[11]

2.7.2 Virtual Reality

Possible interaction interfaces between PksJ ACP4 domain to PksJ KR2 domain were explored in virtual reality as implemented in ChimeraX.[12] The interaction epitope on the PksJ ACP4 domain obtained from the alanine scanning mutagenesis was compared to masked sites in carbene footprinting of PksJ KR2 domain. The distance to the active site, channels leading to the active site, complementarity of interactions on the two domains and shape complementarity were considered. A visual inspection suggested two viable docking interfaces at the two opposite ends of the channel leading to the active site. Viability of the two interaction interfaces was further confirmed through docking simulations.

2.7.3 Protein Docking Simulations

A series of docking simulations for the PksJ ACP4 domain to PksJ KR2 complex were performed using the High Ambiguity Driven protein-protein DOCKing (HADDOCK 2.4) webserver. Simulations were conducted using the PksJ ACP4 domain homology model (see *Section 2.7.1*) and the crystal structure of the PksJ KR2 domain.[13] The 'active' residues for the PksJ ACP4 domain were defined based on results from alanine scanning mutagenesis and the 'active' residues for PksJ KR2 domain were defined as surface residues from peptides masked in the carbene footprinting experiments. In the first instance, all masked residues on the PksJ KR2 domain were used, in addition to a < 15 Å unambiguous distance restrain between S46 of PksJ ACP4 domain and Y386 of the PksJ KR2 domain. This yielded an unreasonable docking solution, with the ACP positioned between the two opposite openings of the channel leading to the active site. In subsequent docking simulations the active residues for PksJ KR2 domain were divided into two groups corresponding to surface residues for masked residues near each of the openings of the channel leading to the active site. Docking was repeated in two different runs using active residues from each of the group together with active residues for PksJ ACP4 domain and an ambiguous restraint of < 15 Å distance between S46 and Y386. Both sets of simulations resulted in reasonable models of the complex that were used as input for choosing residues for mutational analysis of PksJ KR2 domain. The final model of the complex was obtained by using only residues identified in mutational analysis of both interacting partners. The 'active' residues for PksJ ACP4 domain were: E25, R28, I47, I48, Q51, R55, R58. The 'active' residues for PksJ KR2 domain were: R228, M327, D328, E421. A distance of < 15 Å between S439 of the PksJ KR2 domain and S46 of the PksJ ACP4 domain was also used as an unambiguous restraint.

2.7.4 Molecular dynamic simulations

Initial coordinates for simulation of the PksJ ACP4:KR2 domain complex were taken from the first model of the highest scoring cluster from the final docking simulation. Loops missing in the crystal structure of PksJ KR2 were modelled using MODELLER.[14] The Ppant arm was placed manually in virtual reality in ChimeraX. NADPH was modelled in by alignment with $NADP^+$ in the crystal structure of the PksJ KR2 domain. Molecular dynamics (MD) simulations were performed using the AMBER ff19SB force field.[15] The resulting structure was neutralised with Na^+ ions and solvated with OPCBOX water, such that no atom belonging to the complex was less than 10 Å from any box edge, using the TLEAP program.[16] The parameters for NADPH were obtained from AMBER Parameter Database (http://amber.manchester.ac.uk) from deposition by U. Ryde and the parameters for the Ppant arm were obtained from Ray Luo group (http://rayluolab.org/pff-library/).[17] MD heating, equilibration, and production steps were performed using the GPU accelerated AMBER 20 software[16] on a local workstation equipped with Nvidia GeForce 2080 Ti graphics cards. Simulation used the SHAKE algorithm[18] to constrain all protein bonds involving a hydrogen atom; a 2.0 fs time-step was used in these simulations. Long-range electrostatics were calculated using the Particle Mesh Ewald (PME) method with a 12.0 Å cut-off.[19] PME was used for nonbonded interactions. In all simulations, the Langevin thermostat (γ = 2.0 ps^{-1}) was used to maintain temperature control.[20] The solvated protein was then equilibrated by carrying out a short minimization, 50 ps of heating and 50 ps of density equilibration with weak restraints on the protein followed by 500 ps of constant pressure equilibration at 300 K. After a two-step minimization process, in which solvent molecules were allowed to relax before the entire system was minimized, the system was slowly heated to 300 K over 0.1 ns in a canonical ensemble (NVT) simulation, then equilibrated for 2 ns by performing isothermal-isobaric (NPT) simulations at 300K using a Berendsen barostat.[21] A 200 ns production using classic approach (with no acceleration) run was performed with simulation frames written every 10 ps for analysis. Long-range

electrostatics were calculated using the Particle Mesh Ewald (PME) method with an 8.0 Å cutoff. Chimera and ChimeraX were used throughout the course to prepare and visualize the structures. Images of protein structures depicted in **Fig. 4** were rendered using ChimeraX.

The 200 ns MD trajectory stripped of water and ions with frames every 1 ns is available for download at Mendeley Data doi:10.17632/8shpf4mrs6.1.

3. Sequences and Tables

Numbering of amino acids excludes the His-Tag region (highlighted in grey), and correlates to numbering in the main text.

pHis$_6$-PksJ KR2-ACP4

```
MGSSHHHHHH SSGLVPRGSH MASMTGGQQM GRGS-

        10         20         30         40         50         60
SERDKKELVN AIEDRAACFL TKQWSLSPIG SAVPGTRTVA ILCCQETADL AAEVSSYFPN

        70         80         90        100        110        120
HLLIDVSRIE NDQSDIDWKE FDGLVDVIGC GWDDEGRLDW IEWVQRLVEF GHKEGLRLLC

       130        140        150        160        170        180
VTKGLESFQN TSVRMAGASR AGLYRMLQCE YSHLISRHMD AEEVTDHRRL AKLIADEFYS

       190        200        210        220        230        240
DSYDAEVCYR DGLRYQAFLK AHPETGKATE QSAVFPKDHV LLITGGTRGI GLLCARHFAE

       250        260        270        280        290        300
CYGVKKLVLT GREQLPPREE WARFKTSNTS LAEKIQAVRE LEAKGVQVEM LSLTLSDDAQ

       310        320        330        340        350        360
VEQTLQHIKR TLGPIGGVIH CAGLTDMDTL AFIRKTSDDI QRVLEPKVSG LTTLYRHVCN

       370        380        390        400        410        420
EPLQFFVLFS SVSAIIPELS AGQADYAMAN SYMDYFAEAH QKHAPIISVQ WPNWKETGMG

       430        440        450        460        470        480
EVTNQAYRDS GLLSITNSEG LRFLDQIVSK KFGPVVLPAM ANQTNWEPEL LMKRRKPHEG

       490        500        510        520        530        540
GLQEAALQSP PARDIEEADE VSKCDGLLSE TQSWLIDLFT EELRIDREDF EIDGLFQDYG

       550        560        570        580        590        600
VDSIILAQVL QRINRKLEAA LDPSILYEYP TIQRFADWLI GSYSERLSAL FGGRISDASA

P-
```

MW (+HisTag, -NMet) = 70,936 Da

---

pHis$_6$-PksJ KR2

```
MGSSHHHHHH SSGLVPRGSH MASMTGGQQM GRGS-

        10         20         30         40         50         60
SERDKKELVN AIEDRAACFL TKQWSLSPIG SAVPGTRTVA ILCCQETADL AAEVSSYFPN

        70         80         90        100        110        120
HLLIDVSRIE NDQSDIDWKE FDGLVDVIGC GWDDEGRLDW IEWVQRLVEF GHKEGLRLLC

       130        140        150        160        170        180
VTKGLESFQN TSVRMAGASR AGLYRMLQCE YSHLISRHMD AEEVTDHRRL AKLIADEFYS

       190        200        210        220        230        240
DSYDAEVCYR DGLRYQAFLK AHPETGKATE QSAVFPKDHV LLITGGTRGI GLLCARHFAE
```

```
       250        260        270        280        290        300
CYGVKKLVLT GREQLPPREE WARFKTSNTS LAEKIQAVRE LEAKGVQVEM LSLTLSDDAQ

       310        320        330        340        350        360
VEQTLQHIKR TLGPIGGVIH CAGLTDMDTL AFIRKTSDDI QRVLEPKVSG LTTLYRHVCN

       370        380        390        400        410        420
EPLQFFVLFS SVSAIIPELS AGQADYAMAN SYMDYFAEAH QKHAPIISVQ WPNWKETGMG

       430        440        450        460        470
EVTNQAYRDS GLLSITNSEG LRFLDQIVSK KFGPVVLPAM ANQTNWEPEL LMK-
```

MW (+HisTag, -NMet) = 56,483 Da

---

pHis$_6$-PksJ KR1

```
MGSSHHHHHH SSGLVPRGSH MASMTGGQQM GRGS-

        10         20         30         40         50         60
ERLMLEPVWE KQNEEREDED LSYTEHIIVL FETERSVTDS IASHMKDARV ITLNEAVGHI

        70         80         90        100        110        120
AERYQCYMQN IFELLQSKVR KLSAGRIIIQ AIVPLEKEKQ LFAGVSGLFK TAEIEFSKLT

       130        140        150        160        170        180
AQVIEIEKPE EMIDLHLKLK DDSRRPFDKQ IRYEAGYRFV KGWREMVLPS ADTLHMPWRD

       190        200        210        220        230        240
EGVYLITGGA GSLGLLFAKE IANRTGRSTI VLTGRSVLSE DKENELEALR SIGAEVVYRE

       250        260        270        280        290        300
ADVSDQHAVR HLLEEIKERY GTLNGIIHGA GSSKDRFIIH KTNEEFQEVL QPKVSGLLHV

       310        320        330        340        350        360
DECSKDFPLD FFIFFSSVSG CLGNAGQADY AAANSFMDAF AEYRRSLAAS KKRFGSTISF

       370        380        390        400        410        420
NWPLWEEGGM QVGAEDEKRM LKTTGMVPMP TDSGLKAFYQ GIVSDKPQVF VMEGQLQKMK

       430        440
QKLLSAGSKA KRNDQRKADQ DQG
```

MW (+HisTag, -NMet) = 53,556 Da

---

pHis$_6$-PksJ ACP4

```
MGSSHHHHHH SSGLVPRGSH MASMTGGQQM GRGS-

        10         20         30         40         50         60
ADEVSKCDGL LSETQSWLID LFTEELRIDR EDFEIDGLFQ DYGVDSIILA QVLQRINRKL

        70         80         90        100
EAALDPSILY EYPTIQRFAD WLIGSYSERL SALFGGRISD ASAP-
```

MW (+HisTag, -NMet) = 15,217 Da

pHis$_8$-PksJ ACP3a

```
MGHHHHHHHH SSGLVPRGSH MASMTGGQQM GRGS-

        10         20         30         40         50         60
HMASMTGGQQ MGRGSDQRKA DQDQGQTRKL EAALIQMVGA ILI41VNTDDID VNTELSEYGF

        70         80         90        100        110
DSVTFTVFTN KINEKFQLEL TPTIFFEYGS VQSLAEYVVA AYQGEWNQDA TAKG
```

MW (+HisTag, -NMet) = 14,631 Da

Table S1. Primers and associated annealing temperatures for the cloning of PksJ ACP4, ACP3a, KR1, KR2-ACP4, and mutagenic primers to generate PksJ KR2 using PksJ KR2-ACP4 as a template. Restriction enzyme/mutagenic sites are highlighted in bold and underlined.

Plasmid	Forward Primer (5'-3')	Reverse Primer (5'-3')	Temp. (°C)
**pET28a (pHis$_6$) PksJ_KR2-ACP4**	ATA**GGATCC**TCAGAAAGAGACAAAAAAGAACTGG	ATA**CTCGAG**TCAAGGTGCTGATGCATCCGAT	58
**pET28a (pHis$_6$) PksJ_ACP4†**	ATA**GGATCC**GCCGATGAAGTTTCCAAATG	ATA**CTCGAG**TCAAGGTGCTGATGCATCCGAT	56
**pET28a (pHis$_8$) PksJ_ACP3a**	ATA**GGATCC**GATCAGAGAAAGGCGGAT	ATA**CTCGAG**TCAGCCTTTAGCAGTAGCAT	58
**pET28a (pHis$_6$) PksJ_KR1**	ATA**GGATCC**GAACGCTTAATGCTTGAA	ATA**CTCGAG**TCATCCTTGATCCTGATC	54
**pET28a (pHis$_6$) PksJ_KR2**	GCTGATGAAG**TGA**AGGAAACCGCATG	AGTTCCGGCTCCCAGTTT	57

† primers and cloning protocol reported previously in Bellamy-Carter et al.,[22] and have been included for completeness.

Table S2. Primers and associated annealing temperatures for construction of PksJ ACP4 and PksJ ACP3a mutants.

Mutation	Forward Primer (5'-3')	Reverse Primer (5'-3')	Annealing Temp. (°C)
	**PksJ ACP4**		
**S12A**	TGGATTATTA**GCT**GAAACACAGTC	TCACATTTGGAAACTTCATC	58
**E13A†**	ATTATTATCT**GCT**ACACAGTCCTGG	CCATCACATTTGGAAACTTC	56
**Q15A†**	ATCTGAAACA**GCT**TCCTGGCTTATTGATCTGTTTAC	AATAATCCATCACATTTGGAAAC	58
**S16A**	TGAAACACAG**GCT**TGGCTTATTG	GATAATAATCCATCACATTTGG	57
**D20A†**	CTGGCTTATT**GCT**CTGTTTACCG	GACTGTGTTTCAGATAATAATC	57
**T23A**	TGATCTGTTT**GCT**GAAGAGCTGAG	ATAAGCCAGGACTGTGTTTC	61
**E24A†**	TCTGTTTACC**GCT**GAGCTGAGAATAG	TCAATAAGCCAGGACTGTG	59
**E25A†**	GTTTACCGAA**GCT**CTGAGAATAGATC	AGATCAATAAGCCAGGAC	56
**L26A†**	TACCGAAGAG**GCT**AGAATAGATCGTGAAGAC	AACAGATCAATAAGCCAG	57
**R27A†**	CGAAGAGCTG**GCT**ATAGATCGTGAAG	GTAAACAGATCAATAAGCC	56
**I28A†**	AGAGCTGAGA**GCT**GATCGTGAAGAC	TCGGTAAACAGATCAATAAG	56
**D29A†**	GCTGAGAATA**GCT**CGTGAAGACTTC	TCTTCGGTAAACAGATCAATAAG	59
**R30A†**	GAGAATAGAT**GCT**GAAGACTTCGAGATTGACG	AGCTCTTCGGTAAACAGATC	59
**E31A†**	AATAGATCGT**GCT**GACTTCGAGATTG	CTCAGCTCTTCGGTAAAC	57
**D32A†**	AGATCGTGAA**GCT**TTCGAGATTGACG	ATTCTCAGCTCTTCGGTAAAC	59
**F33A†**	TCGTGAAGAC**GCT**GAGATTGACGGG	TCTATTCTCAGCTCTTCG	58
**E34A†**	TGAAGACTTC**GCT**ATTGACGGGTTG	CGATCTATTCTCAGCTCTTC	60
**I35A**	AGACTTCGAG**GCT**GACGGGTTGTTTC	TCACGATCTATTCTCAGC	59
**D36A†**	CTTCGAGATT**GCT**GGGTTGTTTCAG	TCTTCACGATCTATTCTCAG	57
**L38A**	GATTGACGGG**GCT**TTTCAGGATTATGGCG	TCGAAGTCTTCACGATCTATTC	60
**Q40A†**	CGGGTTGTTT**GCT**GATTATGGCG	TCAATCTCGAAGTCTTCAC	57
**D41A†**	GTTGTTTCAG**GCT**TATGGCGTGG	CCGTCAATCTCGAAGTCTTC	63
**Y42A†**	GTTTCAGGAT**GCT**GGCGTGGATTC	AACCCGTCAATCTCGAAG	59
**V44A**	GGATTATGGC**GCT**GATTCGATCATTTTG	TGAAACAACCCGTCAATC	60
**I47A**	CGTGGATTCG**GCT**ATTTTGGCAC	CCATAATCCTGAAACAACC	56
**I48A**	GGATTCGATC**GCT**TTGGCACAGG	ACGCCATAATCCTGAAAC	57
**Q51A†**	CATTTTGGCA**GCT**GTGCTCCAGCGTATAAAC	ATCGAATCCACGCCATAATC	62
**L53A**	GGCACAGGTG**GCT**CAGCGTATAAAC	AAAATGATCGAATCCACG	57

**Q54A†**	ACAGGTGCTC**GCT**CGTATAAACCG	GCCAAAATGATCGAATCC	57
**R55A†**	GGTGCTCCAG**GCT**ATAAACCGCAAATTAG	TGTGCCAAAATGATCGAATC	59
**N57A**	CCAGCGTATA**GCT**CGCAAATTAGAGGC	AGCACCTGTGCCAAAATG	60
**R58A†**	GCGTATAAAC**GCT**AAATTAGAGGCAGCGCTCG	TGGAGCACCTGTGCCAAA	64
**K59A†**	TATAAACCGC**GCT**TTAGAGGCAGCGCTCGATCCATC	CGCTGGAGCACCTGTGCC	69
**E61A†**	CCGCAAATTA**GCT**GCAGCGCTCG	TTTATACGCTGGAGCACC	64
**D65A†**	GGCAGCGCTC**GCT**CCATCGATTC	TCTAATTTGCGGTTTATACGCTGG	67
**P66A†**	AGCGCTCGAT**GCT**TCGATTCTATATG	GCCTCTAATTTGCGGTTTATAC	63
**S67A†**	GCTCGATCCA**GCT**ATTCTATATGAATACC	GCTGCCTCTAATTTGCGG	62
**I68A**	CGATCCATCG**GCT**CTATATGAATACCCG	AGCGCTGCCTCTAATTTG	61
**Y70A†**	ATCGATTCTA**GCT**GAATACCCGACAATTCAAAGGTTCGC	GGATCGAGCGCTGCCTCT	67
**E71A†**	GATTCTATAT**GCT**TACCCGACAATTCAAAGGTTCGC	GATGGATCGAGCGCTGCC	66
**Y72A†**	TCTATATGAA**GCT**CCGACAATTCAAAGGTTCGC	ATCGATGGATCGAGCGCT	62
**Q76A†**	CCCGACAATT**GCT**AGGTTCGCAG	TATTCATATAGAATCGATGGATC	58
**R77A†**	GACAATTCAA**GCT**TTCGCAGATTGGCTGATC	GGGTATTCATATAGAATCGATG	58
**D80A†**	AAGGTTCGCA**GCT**TGGCTGATCG	TGAATTGTCGGGTATTCATATAGAATC	64
**W81A†**	GTTCGCAGAT**GCT**CTGATCGGTTC	CTTTGAATTGTCGGGTATTC	58
	**PksJ ACP3a**		
**I41E**	GGTTGGCGCA**GAA**TTGAAAGTAAAC	ATTTGGATTAAGGCTGCTTC	57
**V67Q**	TACATTCACT**CAA**TTTACAAACAAGATCAACG	ACTGAGTCGAATCCGTAC	59

† primers and cloning protocol reported previously in Bellamy-Carter et al.,[22] and have been included here for completeness.

Table S3. Primers and associated annealing temperatures for construction of PksJ KR2 mutants.

Mutation	Forward Primer (5'-3')	Reverse Primer (5'-3')	Annealing Temp. (°C)
**D429A**	AGCGTATCGG**GCT**AGCGGCTTGT	TGATTTGTCACTTCACCCATAC	63
**E467A**	AACAAACTGG**GCT**CCGGAACTGC	TGATTCGCCATTGCAGGG	62
**E469A**	CTGGGAGCCG**GCT**CTGCTGATGA	TTTGTTTGATTCGCCATTGC	62
**K473A**	ACTGCTGATG**GCT**TGAAGGAAACCGCATGAGG	TCCGGCTCCCAGTTTGTT	64
**R228A**	AGGCGGCACA**GCT**GGCATCGGAC	GTAATGAGAAGTACATGATCTTTC	65
**M327A**	TCTGACGGAT**GCT**GATACGCTGG	CCTGCACAATGAATGACAC	67
**D328A**	GACGGATATG**GCT**ACGCTGGCAT	AGACCTGCACAATGAATGAC	66
**E421A**	CGGTATGGGT**GCT**GTGACAAATC	GTTTCTTTCCAGTTCGGC	66

Table S4. Measured masses of PksJ ACP4 and associated mutants in the *apo*-, *holo*- and AcAc-form.

Mutation	*apo*- Calculated Mass (-NMet) / Da	Observed Mass / Da	*holo*- Calculated Mass (-NMet) / Da	Observed Mass / Da	AcAc- Calculated Mass (-NMet) / Da	Observed Mass / Da
**WT**	15216.92	15217.18	15557.26	15556.37	15641.26	15640.37
**S12A**	15200.92	15199.28	15541.26	15540.41	15625.26	15626.44
**E13A**	15158.88	15158.21	15499.22	15498.87	15583.22	15583.12
**Q15A**	15159.87	15158.29	15500.21	15499.37	15584.21	15585.41
**S16A**	15200.92	15199.48	15541.26	15541.18	15625.26	15625.18
**D20A**	15172.91	15172.34	15513.25	15513.39	15597.25	15598.42
**T23A**	15186.89	15186.32	15527.23	15525.29	15611.23	15612.32
**E24A**	15158.88	15158.31	15499.22	15498.29	15583.22	15584.32
**E25A**	15158.88	15158.34	15499.22	15498.27	15583.22	15583.28
**L26A**	15174.84	15173.26	15515.18	15514.24	15599.18	15600.27
**R27A**	15131.81	15131.28	15472.15	15471.22	15556.15	15557.24
**I28A**	15174.84	15174.29	15515.18	15514.22	15599.18	15600.26
**D29A**	15172.91	15172.32	15513.25	15512.29	15597.25	15598.32
**R30A**	15131.81	15131.27	15472.15	15471.19	15556.15	15557.22
**E31A**	15158.88	15158.31	15499.22	15497.25	15583.22	15582.29
**D32A**	15172.91	15172.34	15513.25	15512.26	15597.25	15598.30
**F33A**	15140.82	15140.29	15481.16	15480.22	15565.16	15566.25
**E34A**	15158.88	15158.29	15499.22	15497.24	15583.22	15584.28
**I35A**	15174.84	15174.26	15515.18	15515.22	15599.18	15600.24
**D36A**	15172.91	15172.33	15513.25	15512.32	15597.25	15598.36
**L38A**	15174.84	15174.28	15515.18	15514.27	15599.18	15600.30
**Q40A**	15159.87	15159.30	15500.21	15499.29	15584.21	15585.32
**D41A**	15172.91	15172.33	15513.25	15512.32	15597.25	15598.36
**Y42A**	15124.82	15124.29	15465.16	15464.29	15549.16	15550.32
**V44A**	15188.86	15188.30	15529.20	15528.28	15613.20	15613.31
**I47A**	15174.84	15173.27	15515.18	15514.28	15599.18	15598.29
**I48A**	15174.84	15174.26	15515.18	15514.28	15599.18	15599.30
**Q51A**	15159.87	15159.29	15500.21	15498.29	15584.21	15583.31
**L53A**	15174.84	15174.25	15515.18	15516.27	15599.18	15600.30
**Q54A**	15159.87	15159.30	15500.21	15499.26	15584.21	15585.29

**R55A**	15131.81	15131.23	15472.15	15470.24	15556.15	15556.27
**N57A**	15173.89	15173.26	15514.23	15513.30	15598.23	15599.34
**R58A**	15131.81	15131.23	15472.15	15471.24	15556.15	15556.15
**K59A**	15159.82	15159.25	15500.16	15499.25	15584.16	15585.28
**E61A**	15158.88	15158.30	15499.22	15498.29	15583.22	15583.32
**D65A**	15172.91	15172.32	15513.25	15512.32	15597.25	15598.35
**P66A**	15190.88	15190.30	15531.22	15530.30	15615.22	15616.33
**S67A**	15200.92	15200.22	15541.26	15540.67	15625.26	15625.21
**I68A**	15174.84	15174.27	15515.18	15514.26	15599.18	15599.29
**Y70A**	15124.82	15124.29	15465.16	15464.36	15549.16	15550.39
**E71A**	15158.88	15158.22	15499.22	15498.53	15583.22	15584.75
**Y72A**	15124.82	15123.29	15465.16	15464.31	15549.16	15550.35
**Q76A**	15159.87	15159.28	15500.21	15499.34	15584.21	15584.37
**R77A**	15131.81	15130.23	15472.15	15471.30	15556.15	15557.34
**D80A**	15172.91	15171.33	15513.25	15521.40	15597.25	15597.42
**W81A**	15101.78	15101.20	15442.12	15442.48	15526.12	15527.14

Table S5. Raw values for PksJ KR2-catalysed ketoreduction activity for all alanine-mutants of PksJ ACP4 and PksJ KR2 mutants. The values are expressed as a percentage of WT PksJ ACP4 or PksJ KR2 activity after 5 min incubation.

PksJ ACP4 Mutation	% Ketoreduction Activity (Relative to WT PksJ ACP4)
S12A	94.29 ± 5.90
E13A	91.35 ± 10.96
Q15A	91.95 ± 9.59
S16A	86.03 ± 14.42
D20A	86.85 ± 11.13
T23A	95.22 ± 8.46
E24A	95.37 ± 3.12
E25A	73.15 ± 4.82
L26A	89.46 ± 4.93
R27A	72.65 ± 9.50
I28A	97.93 ± 1.54
D29A	100.00 ± 2.30
R30A	102.67 ± 1.15
E31A	97.22 ± 2.32
D32A	97.56 ± 3.23
F33A	98.32 ± 1.97
E34A	96.03 ± 1.56
I35A	98.22 ± 4.60
D36A	99.35 ± 3.35
L38A	98.34 ± 4.58
Q40A	97.35 ± 3.97
D41A	98.61 ± 7.20
Y42A	96.84 ± 3.67
V44A	83.01 ± 2.49
I47A	25.31 ± 1.40
I48A	68.24 ± 3.47
Q51A	27.38 ± 2.08
L53A	82.79 ± 6.65
Q54A	96.28 ± 4.86
R55A	70.91 ± 6.74
N57A	93.07 ± 9.67
R58A	58.25 ± 17.45
K59A	87.89 ± 7.49
E61A	89.45 ± 11.96
D65A	87.56 ± 11.52
P66A	87.32 ± 13.31
S67A	96.29 ± 5.66
I68A	82.98 ± 1.28
Y70A	96.15 ± 2.24
E71A	97.83 ± 5.11
Y72A	83.42 ± 2.23
Q76A	92.50 ± 1.53
R77A	87.00 ± 4.47
D80A	94.24 ± 6.51
W81A	88.21 ± 11.77
**PksJ KR2 Mutation**	**% Ketoreduction Activity (Relative to WT PksJ KR2)**
R228A	6.0 ± 0.2
M327A	9.2 ± 1.9
D328A	66.0 ± 1.5
E421A	23.2 ± 2.6
E378A	98.3 ± 2.1
E467A	97.7 ± 2.5
E469A	93.5 ± 2.3
K541A	91.9 ± 1.1

N.B. Arbitrary cut-offs bounds of 80 % and 30 % ketoreduction activity are highlighted in black (> 80 %), orange (80 – 30 %), red (< 30 %) and magenta (< 15 %). Error bars represent ± standard deviation from the mean, where *n* = 3.

Table S6. Statistical analysis of PksJ KR2 domain tryptic peptides from carbene footprinting experiments in the absence of NADPH.

	PksJ KR2		PksJ KR2-ACP4		
**PksJ KR2 Tryptic Peptides**	**Frac. Mod.**	**Std. Dev.**	**Frac. Mod.**	**Std. Dev.**	***P* value**
[7-15]	0.180791	0.011525	0.153979	0.00992	0.0379
[16-22]	0.003111	0.000737	0.002443	0.000553	0.2776
[23-37]	0.007714	0.001173	0.015227	0.000971	0.0010
[38-68]	0.061034	0.008394	0.052482	0.007612	0.2612
[69-79]	0.001535	0.000271	0.001221	4.26E-05	0.1185
[80-97]	0.000406	0.000346	0.000206	6.34E-05	0.3805
[98-106]	0.138996	0.043044	0.109133	0.013715	0.3161
[107-113]	0.029746	0.001091	0.043764	0.001434	0.0002
[118-123]	0.024511	0.000692	0.029268	0.005719	0.2259
[124-134]	0.002947	0.001096	0.00085	0.000236	0.0317
[135-140]	0.04829	0.008818	0.051943	0.00222	0.5249
[146-157]	0.008988	0.000375	0.010053	0.000679	0.0761
[158-168]	0.034738	0.005646	0.009384	0.000544	0.0015
[173-190]	0.105525	0.014264	0.026386	0.006605	0.0010
[208-217]	0.00224	0.000471	0.002005	0.00015	0.4565
[218-228]	0.55463	0.062058	0.233029	0.02975	0.0013
[229-236]	0.439705	0.009038	0.355826	0.035364	0.0164
[247-252]	0.091607	0.009034	0.054584	0.007125	0.0051
[259-263]	0.006788	0.001049	0.005711	0.000783	0.2273
[285-309]	0.389778	0.022079	0.406543	0.035223	0.5233
[348-356]	0.217898	0.019089	0.093805	0.006838	0.0004
[403-415]	0.00801	0.000403	0.007243	0.001038	0.2990
[416-428]	0.268779	0.037726	0.111564	0.006788	0.0021
[429-442]	0.307668	0.023097	0.129218	0.034481	0.0017
[443-450]	0.001144	0.000490	0.00193	0.000343	0.0853
[452-473]	0.382867	0.050558	0.158244	0.062716	0.0085

Table S7. Statistical analysis of PksJ KR2 domain tryptic peptides from carbene footprinting experiments in the presence of NADPH.

	PksJ KR2 + NADPH		PksJ KR2-ACP4 + NADPH		
**PksJ KR2 Tryptic Peptides**	**Frac. Mod.**	**Std. Dev.**	**Frac. Mod.**	**Std. Dev.**	***P* value**
[7-15]	0.21988271	0.022412497	0.173241292	0.01589471	0.0424
[16-22]	0.329738554	0.037013408	0.291756114	0.060917234	0.4083
[23-37]	0.177715615	0.023603537	0.17800565	0.029951719	0.9901
[38-68]	0.106514529	0.025853816	0.074720958	0.015585622	0.1422
[69-79]	0.07304555	0.003586104	0.058969309	0.011970974	0.1228
[80-97]	0.047669776	0.014453348	0.02704364	0.007593917	0.0939
[98-106]	0.198427585	0.09644375	0.119368208	0.010795134	0.2311
[107-113]	0.0411164	0.0023608	0.1030142	0.0346477	0.0367
[118-123]	0.017770249	0.00441106	0.013459068	0.000198658	0.1661
[124-134]	0.167183	0.021227844	0.144238557	0.024745854	0.2898
[135-140]	0.485959303	0.061075894	0.369293811	0.070063204	0.0954
[146-157]	0.433179545	0.054348783	0.386682183	0.044314805	0.3148
[158-168]	0.13095649	0.01714015	0.054635157	0.01554731	0.0046
[173-190]	0.310912264	0.036156113	0.223362223	0.027945399	0.0294
[208-217]	0.093232253	0.004629275	0.086314515	0.00791742	0.2615
[218-228]	0.37711539	0.052815793	0.21342623	0.012635184	0.0064
[229-236]	0.807676555	0.032193717	0.753115582	0.010021435	0.0487
[247-252]	0.0966584	0.008872851	0.073710392	0.005498052	0.0190
[259-263]	0.212302475	0.017760633	0.157882243	0.00420738	0.0067
[285-309]	0.369215362	0.024682988	0.31651371	0.075399596	0.3140
[348-356]	0.369393589	0.031625469	0.286995265	0.038876225	0.0465
[403-415]	0.56677815	0.030381465	0.501084019	0.034878793	0.0697
[416-428]	0.385828495	0.030382709	0.265858677	0.047704233	0.0213
[429-442]	0.267003817	0.005954393	0.185632684	0.023596721	0.0044
[443-450]	0.246943861	0.019680112	0.207190718	0.022891053	0.0847
[452-473]	0.476842759	0.028797282	0.342145435	0.06402449	0.0293

REFERENCES

1 J. C. Aon, R. J. Caimi, A. H. Taylor, Q. Lu, F. Oluboyede, J. Dally, M. D. Kessler, J. J. Kerrigan, T. S. Lewis, L. A. Wysocki and P. S. Patel, *Appl. Environ. Microbiol.*, 2008, **74**, 950–958.

2 D. T. Wagner, J. Zeng, C. B. Bailey, D. C. Gay, F. Yuan, H. R. Manion and A. T. Keatinge-Clay, *Structure*, 2017, **25**, 1045–1055.e2.

3 J. R. Whicher, S. Dutta, D. A. Hansen, W. A. Hale, J. A. Chemler, A. M. Dosey, A. R. H. Narayan, K. Hakansson, D. H. Sherman, J. L. Smith and G. Skiniotis, *Nature*, 2014, **510**, 560–564.

4 A. T. Keatinge-Clay and R. M. Stroud, *Structure*, 2006, **14**, 737–748.

5 Y. Y. Tang, C. Y. Kim, I. I. Mathews, D. E. Cane and C. Khosla, *Proc. Natl. Acad. Sci. U. S. A.*, 2006, **103**, 11124–11129.

6 V. Y. Alekseyev, C. W. Liu, D. E. Cane, J. D. Puglisi and C. Khosla, *Protein Sci.*, 2007, **16**, 2093–2107.

7 D. A. Vander Wood and A. T. Keatinge-Clay, *Proteins*, 2018, **86**, 664–675.

8 L. Manzi, A. S. Barrow, D. Scott, R. Layfield, T. G. Wright, J. E. Moses and N. J. Oldham, *Nat. Commun.*, 2016, E13288.

9 J. Y. Yang, R. X. Yan, A. Roy, D. Xu, J. Poisson and Y. Zhang, *Nat. Methods*, 2015, **12**, 7–8.

10 A. S. Haines, X. Dong, Z. Song, R. Farmer, C. Williams, J. Hothersall, E. Ploskon, P. Wattana-amorn, E. R. Stephens, E. Yamada, R. Gurney, Y. Takebayashi, J. Masschelein, R. J. Cox, R. Lavigne, C. L. Willis, T. J. Simpson, J. Crosby, P. J. Winn, C. M. Thomas and M. P. Crump, *Nat. Chem. Biol.*, 2013, **9**, 685–692.

11 V. B. Chen, W. B. Arendall, J. J. Headd, D. A. Keedy, R. M. Immormino, G. J. Kapral, L. W. Murray, J. S. Richardson, D. C. Richardson and D. C. Richardson, *Acta Crystallogr. D. Biol. Crystallogr.*, 2010, **66**, 12–21.

12 T. D. Goddard, C. C. Huang, E. C. Meng, E. F. Pettersen, G. S. Couch, J. H. Morris and T. E. Ferrin, *Protein Sci.*, 2018, **27**, 14–25.

13 D. T. Wagner, J. Zeng, C. B. Bailey, D. C. Gay, F. Yuan, H. R. Manion and A. T. Keatinge-Clay, *Structure*, 2017, **25**, 1045–1055.

14 B. Webb and A. Sali, *Curr. Protoc. Bioinforma.*, 2016, **2016**, 5.6.1-5.6.37.

15 C. Tian, K. Kasavajhala, K. A. A. Belfon, L. Raguette, H. Huang, A. N. Migues, J. Bickel, Y. Wang, J. Pincay, Q. Wu and C. Simmerling, *J. Chem. Theory Comput.*, 2020, **16**, 528–552.

16 D.A. Case, H.M. Aktulga, K. Belfon, I.Y. Ben-Shalom, S.R. Brozell, D.S. Cerutti, T.E. Cheatham, III, V.W.D. Cruzeiro, T.A. Darden, R.E. Duke, G. Giambasu, M.K. Gilson, H. Gohlke, A.W. Goetz, R. Harris, S. Izadi, S.A. Izmailov, C. Jin, K. Kasavajhala, M.C. Kaymak, E. King, A. Kovalenko, T. Kurtzman, T.S. Lee, S. LeGrand, P. Li, C. Lin, J. Liu, T. Luchko, R. Luo, M. Machado, V. Man, M. Manathunga, K.M. Merz, Y. Miao, O. Mikhailovskii, G. Monard, H. Nguyen, K.A. O'Hearn, A. Onufriev, F. Pan, S. Pantano, R. Qi, A. Rahnamoun, D.R. Roe, A. Roitberg, C. Sagui, S. Schott-Verdugo, J. Shen, C.L. Simmerling, N.R. Skrynnikov, J. Smith, J. Swails, R.C. Walker, J. Wang, H. Wei, R.M. Wolf, X. Wu, Y. Xue, D.M. York, S. Zhao, and P.A. Kollman (2021), Amber 2021, University of California, San Francisco.

17 J. C. Milligan, D. J. Lee, D. R. Jackson, A. J. Schaub, J. Beld, J. F. Barajas, J. J. Hale, R. Luo, M. D. Burkart and S. C. Tsai, *Nat. Chem. Biol.*, 2019, **15**, 669–671.

18 J. P. Ryckaert, G. Ciccotti and H. J. C. Berendsen, *J. Comput. Phys.*, 1977, **23**, 327–341.

19 T. Darden, D. York and L. Pedersen, *J. Chem. Phys.*, 1993, **98**, 10089–10092.

20 R. J. Loncharich, B. R. Brooks and R. W. Pastor, *Biopolymers*, 1992, **32**, 523–535.

21 H. J. C. Berendsen, J. P. M. Postma, W. F. Van Gunsteren, A. Dinola and J. R. Haak, *J. Chem. Phys.*, 1984, **81**, 3684–3690.

22 J. Bellamy-Carter, L. O'Grady, M. Passmore, M. Jenner and N. J. Oldham, *Anal. Sens.*, 2021, **1**, 63–69.
